# Crystal structure of tris­(2-methyl-1*H*-imidazol-3-ium) benzene-1,3,5-tri­carboxyl­ate

**DOI:** 10.1107/S2056989025004748

**Published:** 2025-06-06

**Authors:** Weronika Łukaszczyk, Allegra Lohse, Julia Leibing, Sudem Yildizbas, Irwana Rizvanovic, Simone Techert, Jose de Jesus Velazquez-Garcia

**Affiliations:** a Faculty of Chemistry Jagiellonian University in Kraków Gronostajowa 2 30-387 Kraków Poland; b Gymnasium Altona, Hohenzollernring 57-61, 22763 Hamburg, Germany; c BS 06 Berufliche Schule Chemie, Biologie, Pharmazie, Agrarwirtschaft, Ladenbeker Furtweg 151, 21033 Hamburg, Germany; d Deutsches Elektronen-Synchrotron DESY Notkestr 85 22607 Hamburg Germany; e Institut für Röntgenphysik, Georg-August-Universität Göttingen, Friedrich-Hund-Platz 1, Göttingen, 37077, Germany; Universidad Nacional Autónoma de México, México

**Keywords:** crystal structure, trimesic acid, 2-methyl­imidazolium

## Abstract

The structure of the title salt, 3C_4_H_7_N_2_^+^·C_9_H_3_O_6_^3−^ (**1**), is reported. The compound is formed with three 2-methyl­imidazolium cations and a fully deprotonated trimesic acid. The structure is disordered over two orientations, which were refined using a split model (90.99: 9.01occupancy ratio). Analysis of bond distances and angles reveals the differences and similarities between compound **1** and the previously published 2-methyl-1*H*-imidazol-3-ium 3,5-di­carb­oxy­benzoate structure [Baletska *et al.*, (2023 ▸). *Acta Cryst.* E**79**, 1088–1092] and tris­(2-methyl-1*H*- imidazol-3-ium) 5-carb­oxy­benzene-1,3-di­carboxyl­ate 3,5-di­carb­oxy­benzoate [Asprilla-Herrera *et al.* (2025 ▸). *Acta Cryst.* E**81**, 303–309], as well as the neutral counterparts of the ions [Tothadi *et al.* (2020 ▸). *ACS Appl. Mater. Inter­faces*, **12**, 15588–15594; Hachuła *et al.* (2010 ▸). *J. Chem. Crystallogr.***40**, 201–206]. The crystal packing analysis reveals the formation of hydrogen-bonded two-dimensional networks perpendicular to the [111] vector, where neighbouring planes inter­act *via* extensive π–π stacking.

## Abstract 2

The structure of a 2-methyl-1*H*-imidazol-3-ium-trimesate compound was determined by single-crystal X-ray diffraction. The compound is mixture of protonated and deprotonated mol­ecules.

## 1. Chemical context

Benzene-1,3,5-tri­carb­oxy­lic acid (trimesic acid, H_3_btc) is a planar organic mol­ecule with three negatively ionizable carb­oxy­lic groups. Among its versatile applications, H_3_btc has been used for self-assembled mol­ecular monolayer investigations (MacLeod, 2020 ▸; Ha *et al.*, 2010 ▸; Korolkov *et al.*, 2012 ▸), as well as a surface functionalization agent (Lin *et al.*, 2023 ▸; Chen *et al.*, 2014 ▸; Iancu *et al.*, 2013 ▸). Additionally, it has been used as a building block in the structure of several drug-delivery systems, including dendrimers (Salamończyk, 2021 ▸), polymers (Mat Yusuf *et al.*, 2017 ▸), or hydro­gels (Emani *et al.*, 2023 ▸).

The compound 2-methyl­imidazole (2-mIm) is a heterocyclic aromatic mol­ecule. It has been reported as a surface coating agent (Li *et al.*, 2023 ▸), doping agent (Saghaei *et al.*, 2015 ▸), inter­mediate in the synthesis of several drug compounds, as well as co-ligand in complexes of metal ions with anti-inflammatory compounds, presenting inter­esting bioactive properties (Alisir *et al.*, 2021 ▸; Abuhijleh, 2010 ▸; Nnabuike *et al.*, 2024 ▸).

Both compounds are also widely utilized as organic linkers in the preparation of metal–organic frameworks (MOFs). The 2-mIm acts as an organic linker in the most widely reported zeolitic imidazolate frameworks 8 and 67, ZIF-8 and ZIF-67, (Park *et al.*, 2006 ▸; Banerjee *et al.*, 2008 ▸), while H_3_btc is used in the synthesis of MIL-100 (Férey *et al.*, 2004 ▸) and HKUST-1 (Chui *et al.*, 1999 ▸), to cite a few. Some btc-based MOFs and ZIFs have been used as gas adsorbents and separators, catalysts, and for drug-delivery purposes (Zhong *et al.*, 2018*a* ▸,*b* ▸; Zhao *et al.*, 2024 ▸; Huang *et al.*, 2011 ▸; Song *et al.*, 2024 ▸; Abdelhamid, 2021 ▸).

In previous studies, we have used 2-mIm and H_3_btc to synthesize hexa­aqua­cobalt bis­(2-methyl-1*H*-imidazol-3-ium) tetra­aqua­bis­(benzene-1,3,5-tri­carboxyl­ato-κ*O*)cobalt (Velazquez-Garcia & Techert, 2022 ▸), two Co^II^ mixed-ligand MOFs, mDESY-1 and mDESY-2, (Velazquez-Garcia *et al.*, 2025 ▸), 2-methyl-1*H*-imidazol-3-ium 3,5-di­carb­oxy­benzoate (Baletska *et al.*, 2023 ▸), and tris­(2-methyl-1*H*-imidazol-3-ium) 5-carb­oxy­benzene-1,3-di­carboxyl­ate 3,5-di­carb­oxy­benzoate (Asprilla-Herrera *et al.*, 2025 ▸). Here, we used the same organic compounds to synthesize the title compound.
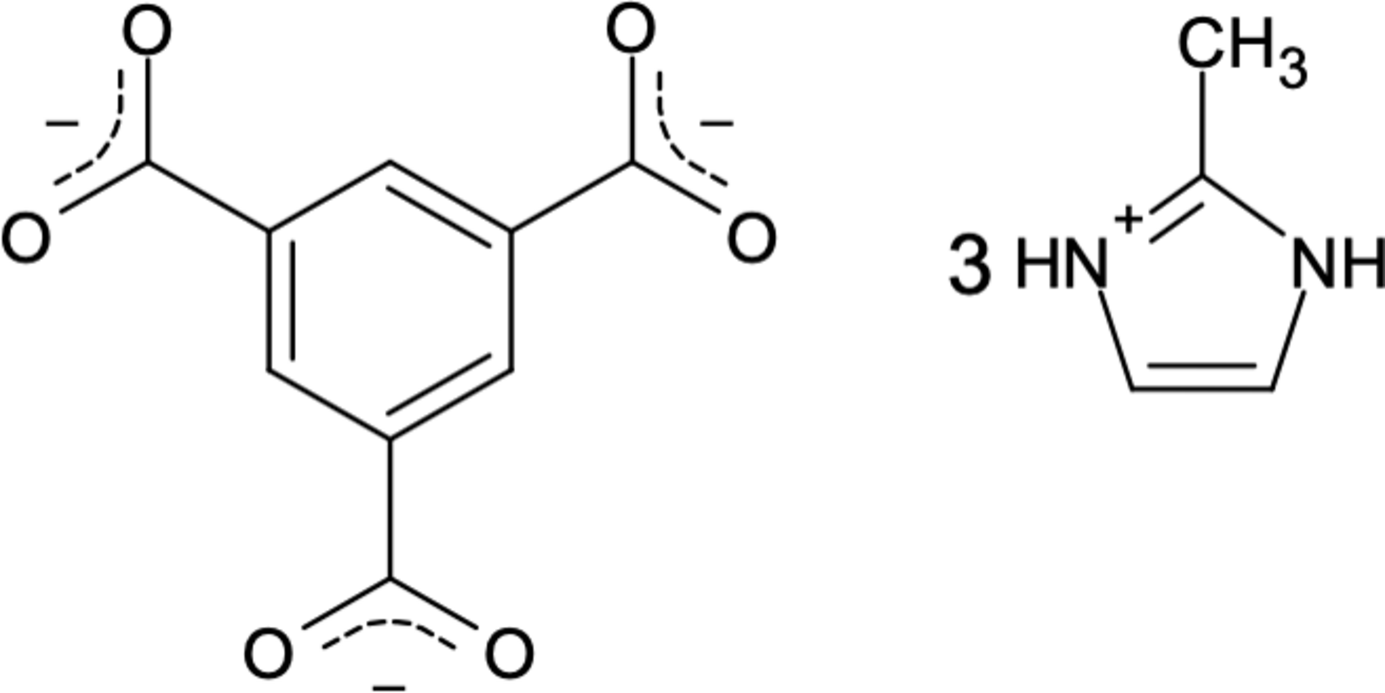


## 2. Structural commentary

**Figure 1:**
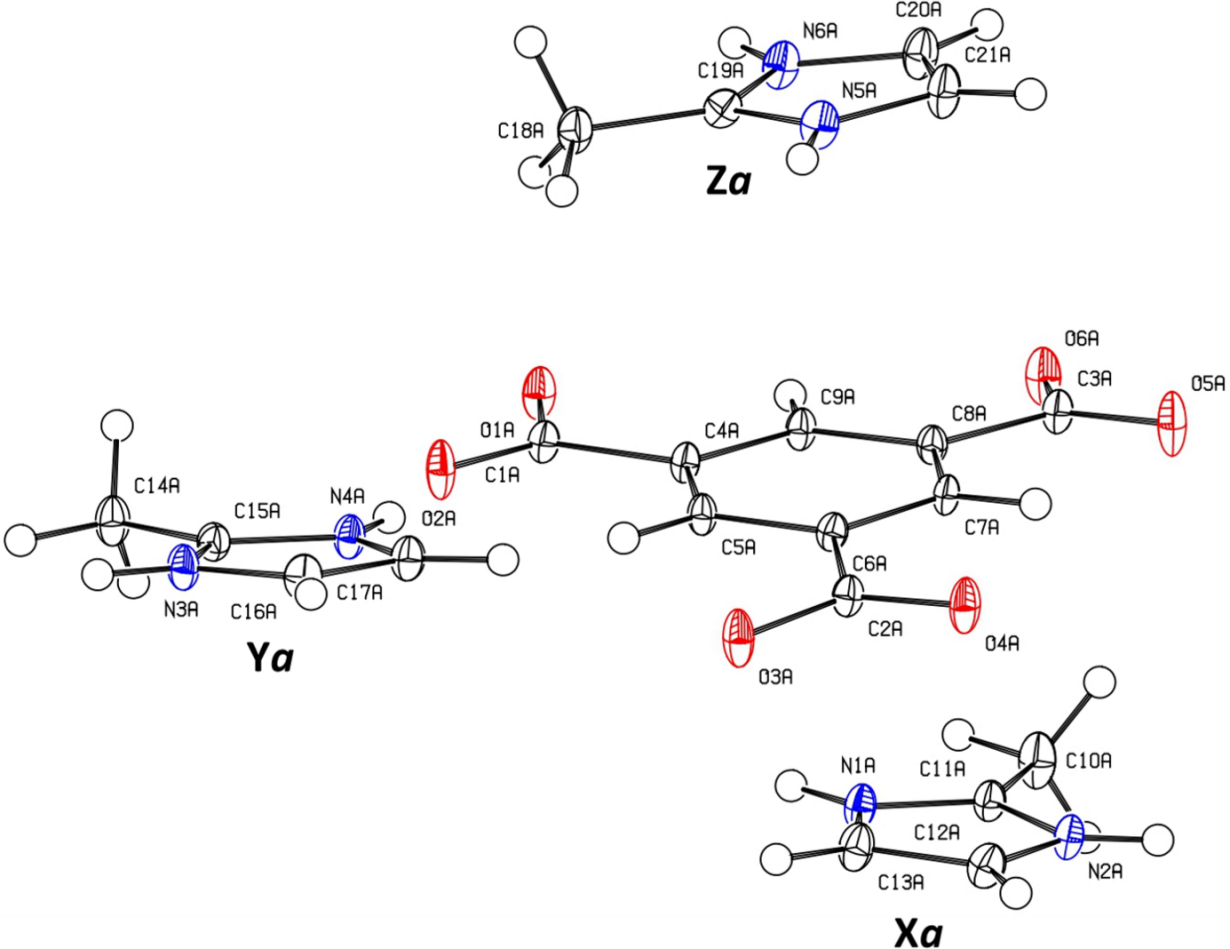
The mol­ecular structure of the major fraction of **1** with displacement ellipsoids drawn at the 50% probability level.

Compound **1** crystallizes in the *P*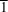 space group. The asymmetric unit comprises one fully deprotonated benzene-1,3,5-tri­carboxyl­ate (btc^3−^) anion and three 2-methyl-1*H*-imidazol-3-ium (H2-mIm^+^) cations. The structure is disordered over two orientations, which were refined using a split model. The major fraction, comprising 90.99%, is labelled *a*, while the minor fraction, comprising 9.01%, is labelled *b*. For clarity, the three H2-mIm^+^ cations were labelled as X*a*, Y*a*, and Z*a* for component *a* and correspondingly as X*b*, Y*b*, and Z*b* for component *b*. The *ORTEP* plot illustrating all ions in the major fraction of **1** is shown in Fig. 1 ▸.

Selected bond lengths, angles, and torsions for the btc^3−^ ions are summarized in Table 1 ▸. In **1***a*, the C—C and C—O bond distances are in the ranges 1.391 (2)–1.516 (2) Å and 1.252 (2)–1.268 (2) Å, respectively. The longest bonds connect carbon atoms of the aromatic ring and carb­oxy­lic groups, with lengths ranging from 1.513 (2) to 1.516 (2) Å. In contrast, the C—C bonds within the aromatic ring are shorter, ranging from 1.391 (2) to 1.395 (2) Å. The highest difference between bond distances within a carb­oxy­lic group is exhibited by the O5—C3—O6 (0.016 Å) and the O3—C2—O4 (0.014 Å) groups. The shortest bond in the structure is formed by C3 and O6 [1.252 (2) Å]. In **1***b*, the C—C bond distances within the aromatic ring are consistent and within the range of 1.38 (2) to 1.39 (2) Å. The C—C bonds between the aromatic ring and carb­oxy­lic groups equal 1.52 (2) Å. The C—O bond distances within carb­oxy­lic groups are consistent, ranging from 1.24 (1) to 1.27 (2) Å. The C—C bond distances range in both **1***a* and **1***b* are similar to the corresponding C—C bond distance ranges in the reported structures of deprotonated H_2_btc^−^ (Baletska *et al.*, 2023 ▸), and H_2_btc^−^ or Hbtc^2−^ (Asprilla-Herrera *et al.*, 2025 ▸), for which they are 1.388 (2)–1.511 (2) Å, 1.389 (2)–1.519 (2) Å, and 1.388 (2)–1.510 (2) Å, respectively. The corresponding C—C bond lengths in the structure of neutral H_3_btc mol­ecule vary slightly less than in **1**, with the range equal to 1.381 (6)–1.494 (9) Å. In contrast, the bond lengths for C—O in both **1***a* and **1***b* are significantly more uniform when compared to the range of 1.229 (5)–1.303 (5) Å for the neutral form (Tothadi *et al.*, 2020 ▸), 1.224 (2)–1.320 (2) Å for H_2_btc^−^ (Baletska *et al.*, 2023 ▸), and 1.214 (2)–1.318 (2) Å and 1.214 (2)–1.338 (2) Å for H_2_btc^−^ and Hbtc^2−^, respectively (Asprilla-Herrera *et al.*, 2025 ▸).

The C—C—C angles in **1** lie within the range 119.0 (2) to 121.0 (1)° for **1***a* and 118 (1)–122 (1)° for **1***b*. These values are consistent with the corresponding angles in H_3_btc [119.0 (4)–121.1 (4)°], H_2_btc^−^ [118.9 (2)–121.4 (4)° (Baletska *et al.*, 2023 ▸) and 118.9 (2)–120.8 (2)° (Asprilla-Herrera *et al.*, 2025 ▸)], and Hbtc^2−^ [119.4 (2)–120 (4)°; Asprilla-Herrera *et al.*, 2025 ▸]. The O—C—O angles in **1***a* fall in the range 124.6 (1)–125.0 (1)° and are comparable to the corresponding angles in H_3_btc [124.4 (4)–125.0 (4)°], singly deprotonated H_2_btc^−^ [123.9 (2)–126.1 (2)° (Baletska *et al.*, 2023 ▸) and 124.3 (2)–126.8 (2)° (Asprilla-Herrera *et al.*, 2025 ▸)], and Hbtc^2−^ [123.2 (2)–125.4 (2)°] forms. In **1***b*, the O—C—O angles are consistent and equal to 121 (1)°.

**Figure 2:**
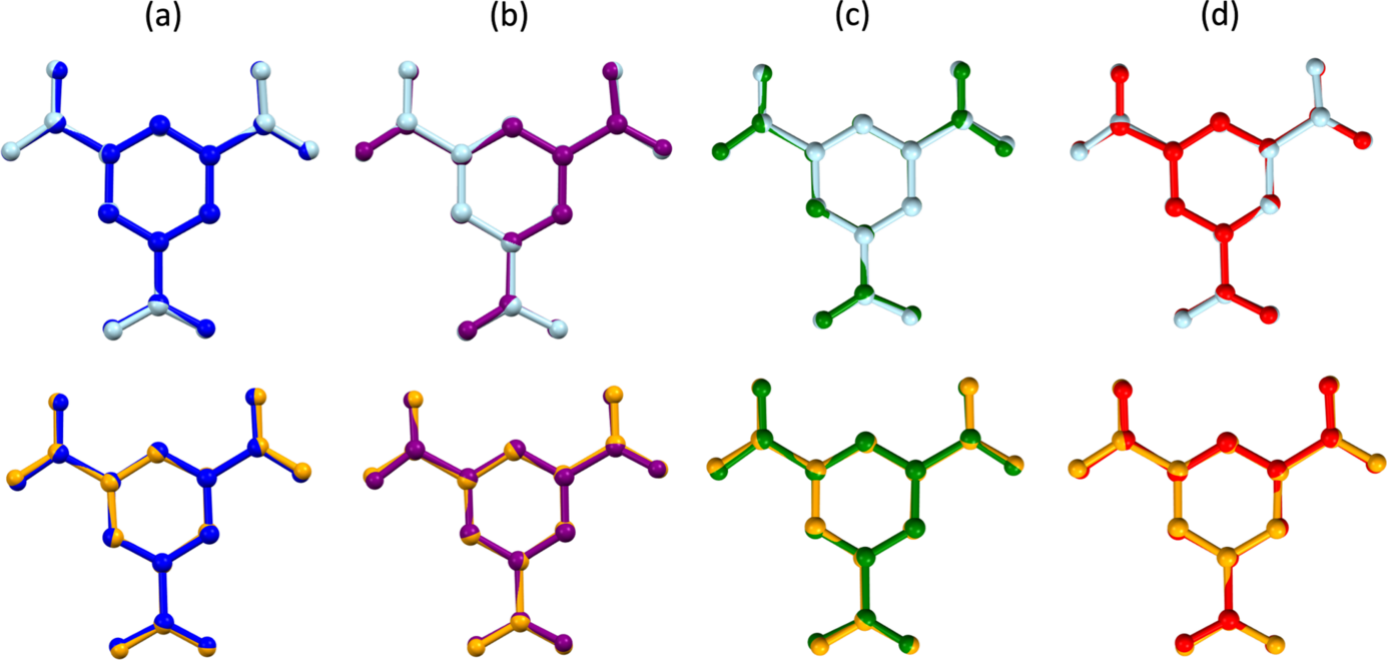
Mol­ecular overlay of btc^3−^ anions from **1***a* (light blue) and **1***b* (orange) with (*a*) neutral H_3_btc mol­ecule (dark blue; Tothadi *et al.*, 2020 ▸), (*b*) H_2_btc^−^ anion (purple; Baletska *et al.*, 2023 ▸), (*c*) H_2_btc^−^ anion (dark green; Asprilla-Herrera *et al.*, 2025 ▸), and (*d*) Hbtc^2−^ (red; Asprilla-Herrera *et al.*, 2025 ▸).

Further comparison of btc^3−^ ions in **1** and the previously reported structures was conducted by analysing the torsion angles and performing mol­ecular overlays. The torsion angles deviation from 0 or 180° are similar for both **1***a* and **1***b* [0.3 (2)–6.8 (1)° and 0 (1)–9 (1)°, respectively]. These values are significantly lower compared to the H_2_btc^−^ structure published by Baletska *et al.* [4.2 (2)–16.6 (2)°] and doubly deprotonated Hbtc^2−^ structure published by Asprilla-Herrera *et al.* [12.6 (2)–17.1 (2)°]. Inter­estingly, in both **1***a* and **1***b*, the torsion angles resemble more the corresponding angles in fully protonated H_3_btc [0 (4)–4.2 (4)°] and singly deprotonated H_2_btc^−^ reported by Asprilla-Herrera *et al.* [0.6 (2)–7.0 (2)°]. This is further corroborated by mol­ecular overlays of the btc^3−^ ions with other reported structures of btc (Fig. 2 ▸) and their respective root-mean-squared deviation (r.m.s.d.) and maximal deviation (max. d.) values (Table 2 ▸) generated with the *Mercury* software (Macrae *et al.*, 2020 ▸). The r.m.s.d and max. d. values calculated for mol­ecular overlays of btc^3−^ of **1***a* and **1***b* with H_3_btc and H_2_btc^−^ (Asprilla-Herrera *et al.*, 2025 ▸) are notably lower (with the largest r.m.s.d. equal to 0.0776 Å and max. d. equal to 0.1626 Å for btc^−^ of **1***b* overlayed with H_2_btc^−^ reported by Asprilla-Herrera *et al.*) compared to the overlays with the other reported btc structures (with the lowest r.m.s.d. equal to 0.1067 Å and max. d. equal to 0.2231 Å for **1***b* overlayed with H_2_btc^−^ reported by Baletska *et al.*). Note that hydrogen atoms were excluded from the calculation.

Table 3 ▸. presents selected bond lengths, angles, and torsions for the H2-mIm^+^ cations. The corresponding C—C and C—N bond distances are rather uniform across the individual cations. The C—C bond distances fall in the range of 1.349 (2)–1.480 (2) Å for **1***a* and 1.34 (2)–1.48 (2) Å for **1***b*. The C—N bond distances vary from 1.323 (2) to 1.382 (3) Å for **1***a* and from 1.31 (2) to 1.40 (3) Å for **1***b*. The distances of both aromatic C—C bonds and C—C bonds between the ring and the methyl group of H2-mIm^+^ cations of **1** are more similar to those observed in the structures of H2-mIm^+^ cations reported by Baletska *et al.* [1.345 (3) and 1.481 (3) Å, respectively] and Asprilla-Herrera *et al.* [ion a: 1.348 (2) and 1.483 (3) Å, respectively] than the neutral 2-mIm structure published by Hachuła *et al.*, 2010 ▸ [1.367 (1) Å and 1.488 (1) Å, respectively]. Only a slight asymmetry of endocyclic N—C bonds was observed for the X*a* and Z*a* H2-mIm^+^ cations in the structure of **1**, suggesting a greater double-bond character of the N1—C11 and N5—C19 bonds than the N2—C11 and N6—C19 bonds, accordingly. In **1***a*, the difference in distance is comparable (from 0.004 to 0.008 Å) to that of the structure from Baletska *et al.* (0.008 Å), and for **1***b* (from 0.02–0.03 Å), it is similar to that of the structure of the neutral 2-mIm mol­ecule (0.022 Å).

Similar to other H2-mIm^+^ structures, protonation introduces more symmetry regarding the bond angles within the aromatic ring. The largest deviation from the ideal penta­gon inter­ior angle of 108° is 1.7° in fraction **1***a* (X*a* ion) and 4° in fraction **1***b* (X*b* ion). In comparison, the corresponding deviation in the structure of the neutral 2-mIm form is 3.4°. The methyl groups in cations of **1** show the maximal deviation from coplanarity with the aromatic ring in the Z*a* (1.9°) and X*b* (3°) ions. Compared to other reported structures, these values are the closest to those reported by Asprilla-Herrera *et al.* in one of the ions in the structure (for which the maximal deviation reported was 2.3°). In the other 2-mIm^+^ and 2-mIm structures, the corresponding maximal deviation from planarity was no higher than 0.9°.

**Figure 3:**
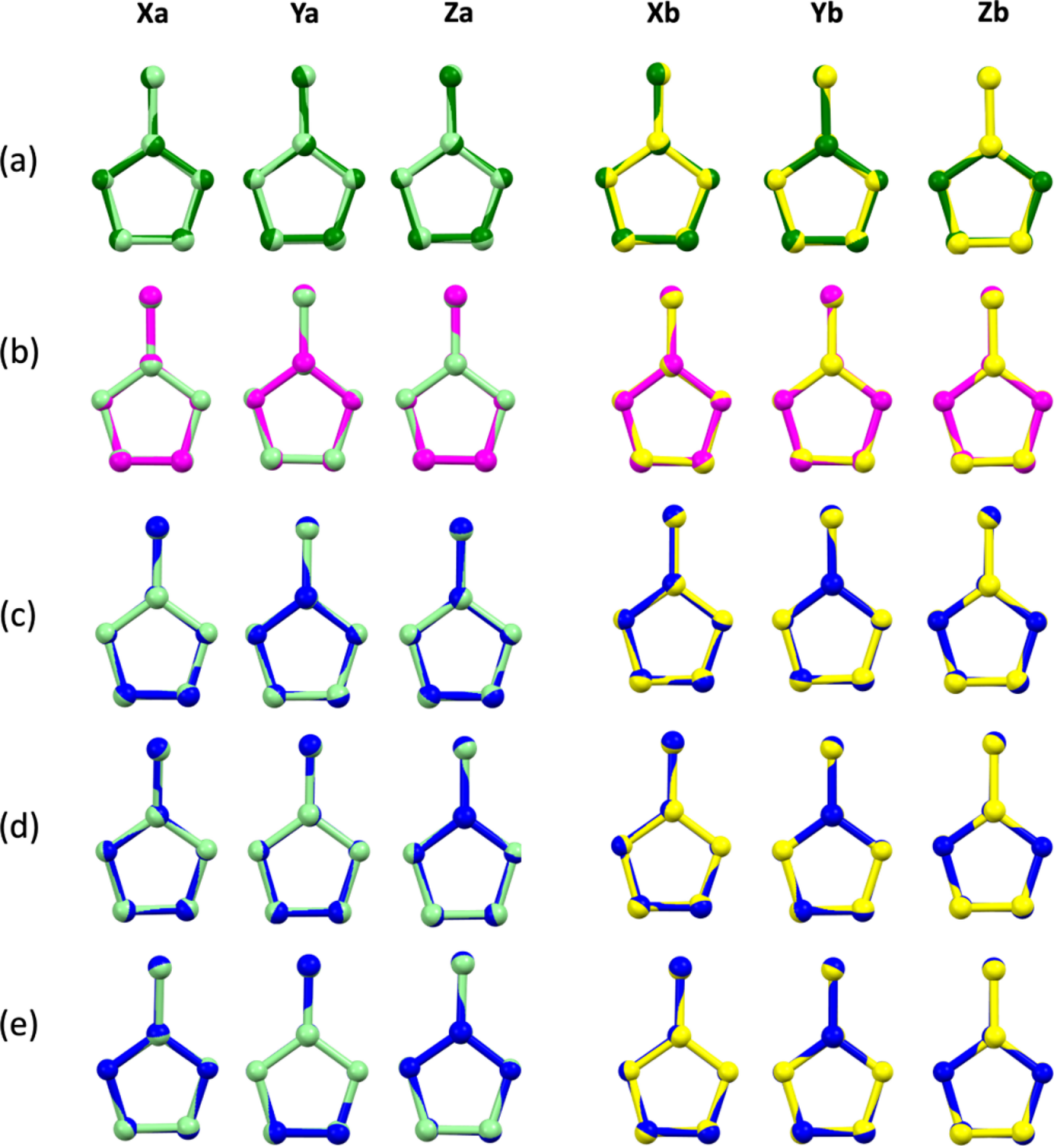
Mol­ecular overlay plot of H2-mIm^+^ cations of **1***a* (light green) and **1***b* (yellow) with (*a*) neutral 2-mIm mol­ecule (dark green; Hachuła *et al.*, 2010 ▸), (*b*) H2-mIm^+^ cation (magenta; Baletska *et al.*, 2023 ▸) and H2-mIm^+^ cations adapted from Asprilla-Herrera *et al.* (blue; *c*, *d*, *e* – cations *A*, *B*, and *C*, respectively).

The values of root-mean-squared deviation (r. m. s. d.) and maximal deviation (max. d.) values calculated by *Mercury* software for the mol­ecular overlays of H2-mIm^+^ cations of **1** with the neutral H2-mIm mol­ecule and the other H2-mIm^+^ cations are presented in Table 4 ▸. The mol­ecular overlays are depicted in Fig. 3 ▸. The values suggest a higher resemblance of H2-mIm^+^ cations of **1** to other reported protonated forms, with the lowest value of r.m.s.d. and max. d. recorded for the overlay of X*a* with the B ion from the structure reported by Asprilla-Herrera *et al.* (0.0050 and 0.0076 Å, respectively).

## 3. Supra­molecular features

**Figure 4:**
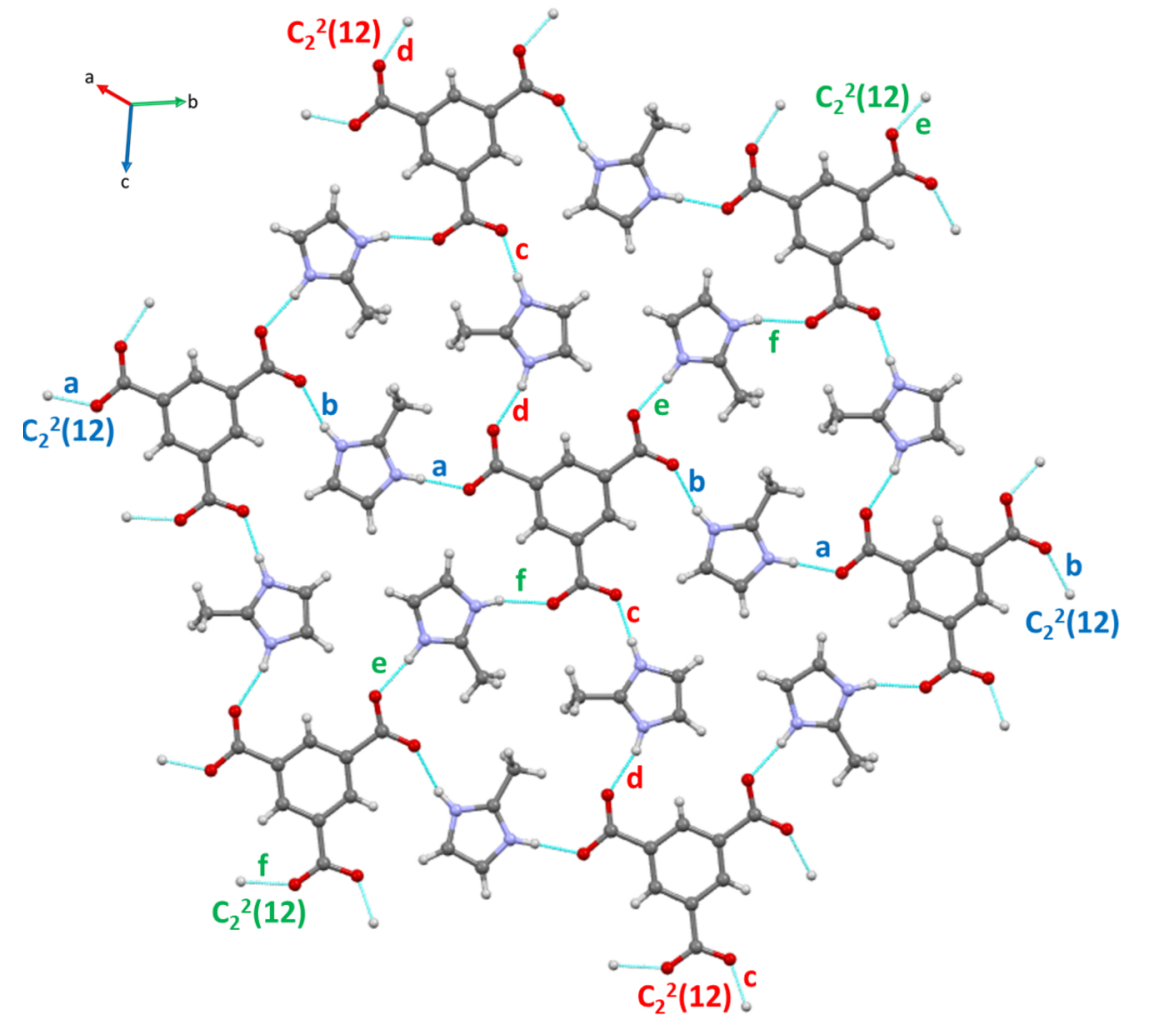
View along the [111] vector showing a network of hydrogen bonds between btc^3−^ and H2-mIm^+^ ions in the *a* fraction. The first-level graph-set descriptors are labelled with letters *a*–*f* (see Table 6 ▸). The colour coding indicates the direction of the 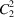
(12) chains of the second-level graph-set descriptors.

**Figure 5:**
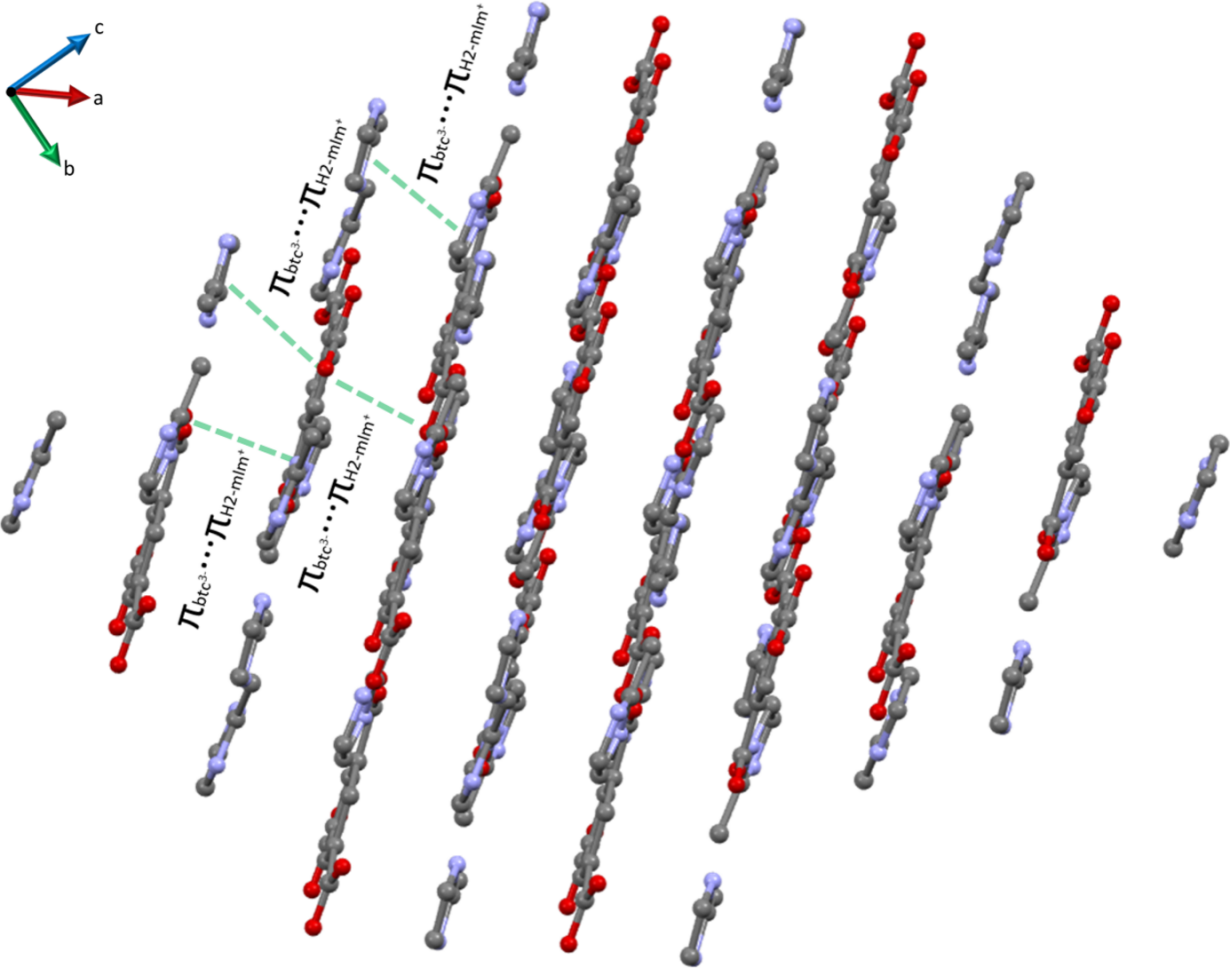
Crystal packing in compound **1** viewed along the [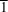
11] vector illustrating the stacking of the planes *via* π–π inter­actions (green lines).

The primary inter­molecular inter­actions contributing to the crystal packing include hydrogen bonds and π–π stacking. The hydrogen bonds form 2D network planes perpendicular to the [111] vector (Fig. 4 ▸), while the π–π stacking between the aromatic rings hold the planes together (Fig. 5 ▸). Table 5 ▸ displays the details of the π–π inter­actions between the planes, while Table 6 ▸ summarizes the geometrical details of the hydrogen-bond network. Note that half of the hydrogen bonds are charge-assisted and therefore, display an ionic character, confirmed by significantly shorter distances between acceptor and donor atoms (Mayer *et al.*, 1992 ▸).

To gain a deeper understanding of the inter­molecular inter­action patterns within **1**, a graph-set analysis (Etter *et al.*, 1990 ▸; Bernstein *et al.*, 1995 ▸) was performed. The analysis reveals that **1** contains only six discrete *D*(2) motifs at the first-level graph set. The second-level graph set features three 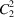
(12) and twelve *D*_2_^2^ (Table 7 ▸) motifs. No other types of patterns were identified during the graph-set analysis.

## 4. Hirshfeld surface analysis

**Figure 6:**
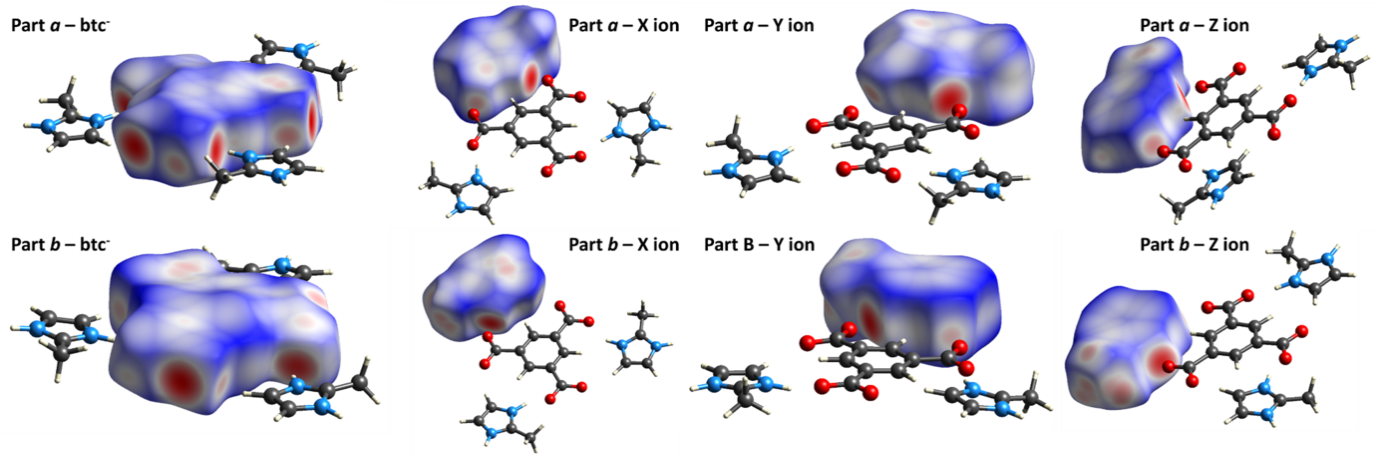
Hirshfeld surface for each ion in both fractions of **1** mapped over *d*_norm_.

Inter­molecular inter­actions in both fractions of **1** were further qu­anti­fied using Hirshfeld surface analysis with *CrystalExplorer 17.5* (Turner *et al.* 2017 ▸). The three-dimensional *d*_norm_ surfaces were plotted with a standard resolution and a fixed colour scale ranging from −0.7640 (red) to 1.0884 (blue) a.u. for fraction *a* and from −0.8458 (red) to 1.0400 (blue) for the minor fraction *b*. The pale-red spots in Fig. 6 ▸ indicate short contacts and negative *d*_norm_ values on the surface, corresponding to the inter­actions previously described.

**Figure 7:**
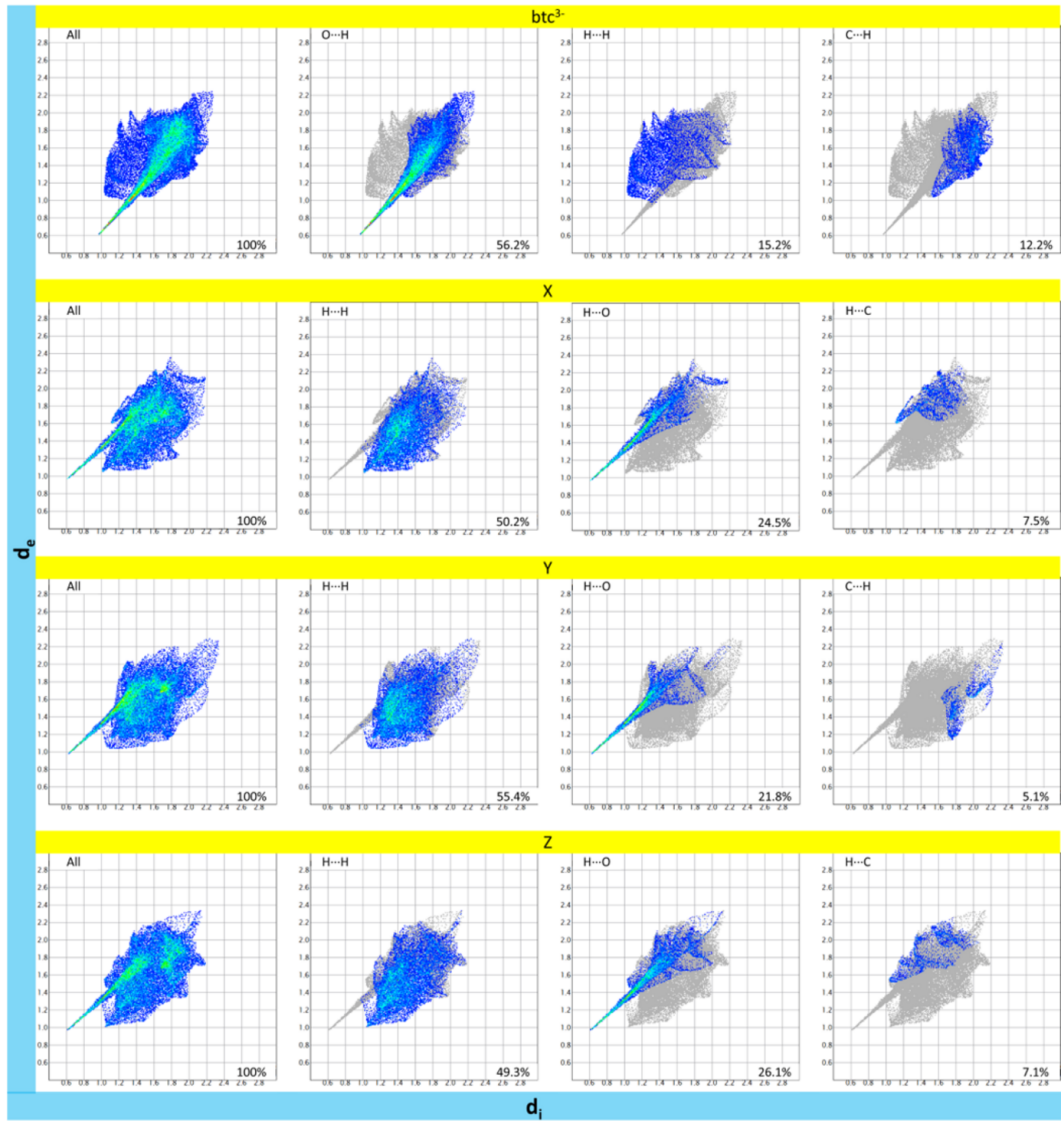
Fingerprint plots of the Hirshfeld surfaces for fraction *a* of **1**, showing the overall plot and three most significant inter­molecular contributions.

**Figure 8:**
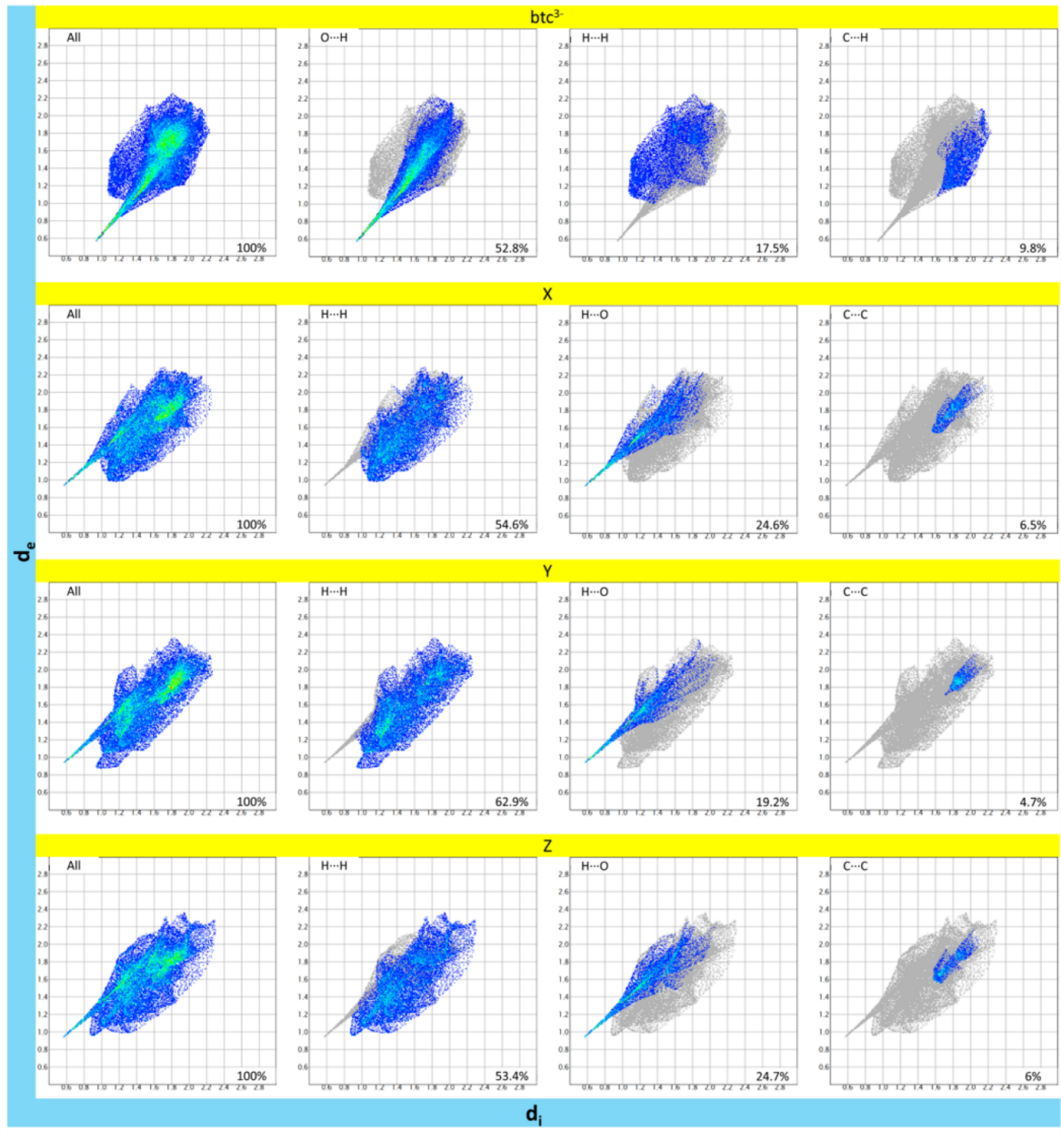
Fingerprint plots of the Hirshfeld surfaces for fraction *b* of **1**, showing the overall plot and three most significant inter­molecular contributions.

The two-dimensional fingerprint plots for fractions *a* and *b* are illustrated in Fig. 7 ▸ and Fig. 8 ▸, respectively, with the contributions per inter­action per ion summarized in Table 8 ▸. In both fractions, the greatest contributions for the btc^3−^ ions are O⋯H (>50%) and H⋯H (> 15%), while for the three H2-mIm^+^ ions are the H⋯H (> 50%) and H⋯O (> 19%).

## 5. Database survey

No reported structures of the title compound were found in the Cambridge Structural Database (CSD version 5.45, update of November 2023; Groom *et al.*, 2016 ▸). The closest to **1** are the previously mentioned structures reported under the refcodes ZUQYOD (Asprilla-Herrera *et al.*, 2025 ▸) and LODSUW (Baletska *et al.*, 2023 ▸).

Some structures containing H2-mIm^+^ cation were reported under the refcodes BEZGEU (Dhanabal *et al.*, 2013 ▸), BOTTEK, BOTTIO, BOTTOU (Meng *et al.*, 2009 ▸), BOTTEK01, BOTTIO01, BOTTOU01, VURBUG, VURCAN, VURFAQ (Callear *et al.*, 2010 ▸), DAMGIL (Hinokimoto *et al.*, 2021 ▸), DOWVUI (Shi *et al.*, 2014 ▸), FAMFIL, FAMFOR, FAMFUX (Zhang & Zhang, 2017 ▸), FETDAK (Aakeröy *et al.*, 2005 ▸), HILSOL (Qu, 2007 ▸). However, these structures do not have the btc^3−^ ion.

Among the various reported structures containing fully deprotonated btc^3−^ ion with other organic cations, we highlight those with the following refcodes: HEGFOQ (Zhu *et al.*, 2011 ▸), HOPZIX (Ndoye *et al.*, 2013 ▸), IJEQIX (Lynch, 2003 ▸), LIDHIT, LIDJIV (Skala *et al.*, 2023 ▸), MEKKES, MEKKIW, MEKKOC (Plaut *et al.*, 2000 ▸), OSADOD (Singh *et al.*, 2016 ▸), OTINUB (Gupta *et al.*, 2011 ▸), TOZZUD, TUBBAT (Melendez *et al.*, 1996 ▸), VABQOG (Liu *et al.*, 2010 ▸), and WONVAX (Hayashi *et al.*, 2008 ▸). However, these structures do not contain the H2-mIm^+^ cation.

Some compounds with low resemblance to the title compound were reported under the refcodes CUMQUX (Basu *et al.*, 2009 ▸), HICSUJ (Lie *et al.*, 2013 ▸), ILELAO (Li & Li, 2016 ▸), JOCBAH (Falek *et al.*, 2019 ▸), LUBGUM, LUBHAT, LUBHEX, LUBHIB, LUBHOH, LUBHUN, LUBJAV (Singh *et al.*, 2015 ▸), SUHRAR (Rajkumar *et al.*, 2020 ▸), YOCSIT (Habib & Janiak, 2008 ▸), WOGBED (Sosa-Rivadeneyra *et al.*, 2024 ▸).

## 6. Synthesis and crystallization

To synthesize the title compound, 120 µl of a 1.58 *M* ethano­lic solution of 2-mIm was diluted with 2 ml of ethanol, followed by the addition of 100 µl of a 0.12 *M* ethano­lic solution of H_3_btc. The mixture was gently shaken and left to rest at 313 K. After one week, crystals of **1** were obtained.

## 7. Refinement

Crystal data, data collection and structure refinement details are summarized in Table 9 ▸. The structure is disordered over two orientations and was refined using a split model with restraint on bond lengths (SADI). SIMU and RIGU restraints were then applied across the minor fraction *b*. Constraints on the atomic displacement parameter (EADP) were also applied to C18*B*, C10*B*, C6*B*, C7*B*, N5*B*, and O6*B* of the minor component, with close by part a atoms. The most disagreeable reflection (1 0 5), with an error/s.u. of more than 10, was omitted using the OMIT instruction in *SHELXL* (Sheldrick, 2015*b* ▸). The positions of hydrogen atoms were refined with *U*_iso_(H) = 1.2*U*_eq_(C or N) for CH and NH groups and *U*_iso_(H) = 1.5*U*_eq_(C or O) for others. Hydrogen atoms attached to nitro­gen atoms were refined with DFIX 0.86 0.01 instruction for the major component, while the HFIX command was applied for the minor component.

## Supplementary Material

Crystal structure: contains datablock(s) I. DOI: 10.1107/S2056989025004748/jq2040sup1.cif

Structure factors: contains datablock(s) I. DOI: 10.1107/S2056989025004748/jq2040Isup2.hkl

Supporting information file. DOI: 10.1107/S2056989025004748/jq2040Isup3.cml

CCDC reference: 2453953

Additional supporting information:  crystallographic information; 3D view; checkCIF report

## Figures and Tables

**Table 1:** Selected bond lengths (Å), angles (°), and torsion angles (°) of the btc^3−^ ions

**1** *a*		**1** *b*	
C4—C5	1.392 (2)	C4—C5	1.38 (2)
C5—C6	1.394 (2)	C5—C6	1.38 (2)
C6—C7	1.395 (2)	C6—C7	1.38 (2)
C7—C8	1.391 (2)	C7—C8	1.38 (2)
C8—C9	1.395 (2)	C8—C9	1.39 (2)
C9—C4	1.394 (2)	C9—C4	1.38 (2)
C1—C4	1.513 (2)	C1—C4	1.52 (2)
C2—C6	1.516 (2)	C2—C6	1.52 (1)
C3—C8	1.516 (2)	C3—C8	1.52 (2)
C1—O1	1.263 (2)	C1—O1	1.27 (1)
C1—O2	1.257 (2)	C1—O2	1.25 (2)
C2—O3	1.267 (2)	C2—O3	1.27 (2)
C2—O4	1.253 (2)	C2—O4	1.24 (1)
C3—O5	1.268 (2)	C3—O5	1.27 (2)
C3—O6	1.252 (2)	C3—O6	1.25 (1)
C4—C5—C6	121.0 (1)	C4—C5—C6	122 (1)
C5—C6—C7	119.0 (1)	C5—C6—C7	119 (1)
C6—C7—C8	121.0 (1)	C6—C7—C8	121 (1)
C7—C8—C9	119.1 (1)	C7—C8—C9	118 (1)
C8—C9—C4	120.8 (1)	C8—C9—C4	122 (1)
C9—C4—C5	119.1 (1)	C9—C4—C5	118 (1)
O1—C1—O2	124.9 (1)	O1—C1—O2	121 (1)
O3—C2—O4	125.0 (1)	O3—C2—O4	121 (1)
O5—C3—O6	124.6 (1)	O5—C3—O6	121 (1)
C5—C4—C1—O1	179.4 (1)	C5—C4—C1—O1	171 (1)
C5—C4—C1—O2	1.2 (2)	C5—C4—C1—O2	−4 (2)
C9—C4—C1—O1	−0.3 (2)	C9—C4—C1—O1	−4 (2)
C9—C4—C1—O2	−178.4 (1)	C9—C4—C1—O2	−180 (1)
C5—C6—C2—O3	4.3 (2)	C5—C6—C2—O3	−1 (2)
C5—C6—C2—O4	−176.8 (1)	C5—C6—C2—O4	−178 (1)
C7—C6—C2—O3	−173.2 (1)	C7—C6—C2—O3	−180 (1)
C7—C6—C2—O4	5.7 (2)	C7—C6—C2—O4	3 (2)
C7—C8—C3—O5	−4.4 (2)	C7—C8—C3—O5	2 (2)
C7—C8—C3—O6	174.7 (1)	C7—C8—C3—O6	−174 (1)
C9—C8—C3—O5	177.3 (1)	C9—C8—C3—O5	−179 (1)
C9—C8—C3—O6	−3.7 (2)	C9—C8—C3—O6	6 (2)

**Table 2:** Root-mean-square-deviation and maximal deviation values calculated for mol­ecular overlays of btc^3−^ in **1** and other reported btc structures

	**1** *a*		**1** *b*	
	r.m.s.d	max. d.	r.m.s.d.	max. d.
H_3_btc (Tothadi *et al.*, 2020 ▸)	0.0695	0.1509	0.0643	0.1098
H_2_btc^−^ (Baletska *et al.*, 2023 ▸)	0.1067	0.2231	0.1383	0.3149
H_2_btc^−^ (Asprilla-Herrera *et al.*, 2025 ▸)	0.0592	0.1135	0.0776	0.1626
Hbtc^2−^ (Asprilla-Herrera *et al.*, 2025 ▸)	0.1522	0.3301	0.1804	0.3712

**Table 3:** Selected bond lengths (Å), angles (°), and torsion angles (°C) of the H2-mIm^+^ ions

X*a*		Y*a*		Z*a*	
C10—C11	1.480 (2)	C14—C15	1.476 (2)	C18—C19	1.473 (2)
C12—C13	1.349 (2)	C16—C17	1.352 (2)	C20—C21	1.349 (2)
N1—C11	1.323 (2)	N3—C15	1.329 (2)	N5—C19	1.324 (2)
N1—C13	1.376 (2)	N3—C16	1.375 (2)	N5—C21	1.378 (2)
N2—C11	1.331 (2)	N4—C15	1.333 (3)	N6—C19	1.336 (3)
N2—C12	1.381 (3)	N4—C17	1.380 (3)	N6—C20	1.382 (3)
C10—C11—N1	125.1 (1)	C14—C15—N3	125.3 (1)	C18—C19—N5	125.3 (1)
C11—N1—C13	108.5 (1)	C15—N3—C16	108.7 (1)	C19—N5—C21	108.6 (1)
N1—C13—C12	107.8 (1)	N3—C16—C17	107.5 (1)	N5—C21—C20	107.6 (2)
C13—C12—N2	106.3 (2)	C16—C17—N4	106.5 (2)	C21—C20—N6	106.6 (2)
C12—N2—C11	108.9 (2)	C17—N4—C15	109.0 (2)	C20—N6—C19	108.7 (2)
N2—C11—N1	108.6 (2)	N4—C15—N3	108.3 (2)	N6—C19—N5	108.5 (2)
N2—C11—C10	126.3 (2)	N4—C15—C14	126.4 (2)	N6—C19—C18	126.1 (2)
C13—N1—C11—C10	179.5 (2)	C16—N3—C15—C14	−178.4 (2)	C21—N5—C19—C18	178.3 (2)
C12—N2—C11—C10	−179.2 (2)	C17—N4—C15—C14	178.5 (2)	C20—N6—C19—C18	−178.1 (2)
					
X*b*		Y*b*		Z*b*	
C10—C11	1.48 (2)	C14—C15	1.47 (2)	C18—C19	1.47 (2)
C12—C13	1.34 (2)	C16—C17	1.35 (2)	C20—C21	1.34 (2)
N1—C11	1.31 (2)	N3—C15	1.32 (2)	N5—C19	1.32 (1)
N1—C13	1.36 (2)	N3—C16	1.36 (2)	N5—C21	1.37 (2)
N2—C11	1.34 (2)	N4—C15	1.34 (4)	N6—C19	1.34 (3)
N2—C12	1.39 (3)	N4—C17	1.39 (2)	N6—C20	1.40 (3)
C10—C11—N1	126 (1)	C14—C15—N3	126 (1)	C18—C19—N5	127 (1)
C11—N1—C13	108 (1)	C15—N3—C16	108 (1)	C19—N5—C21	108 (1)
N1—C13—C12	110 (1)	N3—C16—C17	109 (1)	N5—C21—C20	109 (1)
C13—C12—N2	104 (2)	C16—C17—N4	105 (2)	C21—C20—N6	105 (1)
C12—N2—C11	109 (2)	C17—N4—C15	109 (2)	C20—N6—C19	109 (2)
N2—C11—N1	109 (2)	N4—C15—N3	108 (2)	N6—C19—N5	108 (1)
N2—C11—C10	125 (2)	N4—C15—C14	125 (2)	N6—C19—C18	124 (2)
C13—N1—C11—C10	−177 (2)	C16—N3—C15—C14	178 (1)	C21—N5—C19—C18	179 (1)
C12—N2—C11—C10	178 (2)	C17—N4—C15—C14	−179 (2)	C20—N6—C19—C18	−178 (2)

**Table 4:** Root-mean-square-deviation and maximal deviation values calculated for mol­ecular overlays of H2-mIm^+^ ions in **1** and other reported 2-mIm structures

	X*a*		Y*a*		Z*a*	
	r.m.s.d.	max. d.	r.m.s.d.	max. d.	r.m.s.d.	max. d.
2-mIm (Hachuła *et al.*, 2010 ▸)	0.0269	0.0430	0.0268	0.0430	0.0268	0.0385
H2-mIm^+^ (Baletska *et al.*, 2023 ▸)	0.0102	0.0125	0.0093	0.0123	0.0141	0.0202
H2-mIm^+^ ion *A* (Asprilla-Herrera *et al.*, 2025 ▸)	0.0123	0.0167	0.0094	0.0143	0.0111	0.0169
H2-mIm^+^ ion *B* (Asprilla-Herrera *et al.*, 2025 ▸)	0.0050	0.0076	0.0064	0.0097	0.0075	0.0108
H2-mIm^+^ ion *C* (Asprilla-Herrera *et al.*, 2025 ▸)	0.0075	0.0104	0.0091	0.0120	0.0103	0.0157
						
	X*b*		Y*b*		Z*b*	
2-mIm (Hachuła *et al.*, 2010 ▸)	0.0265	0.0409	0.0298	0.0451	0.0419	0.0612
H2-mIm^+^ (Baletska *et al.*, 2023 ▸)	0.0216	0.0352	0.0233	0.0390	0.0368	0.0468
H2-mIm^+^ ion *A* (Asprilla-Herrera *et al.*, 2025 ▸)	0.0214	0.0351	0.0255	0.0462	0.0331	0.0437
H2-mIm^+^ ion *B* (Asprilla-Herrera *et al.*, 2025 ▸)	0.0178	0.0317	0.0203	0.0372	0.0309	0.0431
H2-mIm^+^ ion *C* (Asprilla-Herrera *et al.*, 2025 ▸)	0.0237	0.0404	0.0227	0.0342	0.0359	0.0470

**Table 5:** Geometrical details of *π*–*π* inter­actions (Å) in **1**

Ion	H2-mIm^−^	Centroid-to-centroid distance	Perpendicular distance	Offset
btc^3−^ (**1***a*)	X*a*	3.6855 (10)	3.3	1.629
btc^3−^ (**1***a*)	Z*a*	3.8392 (12)	3.4	1.771
X*a*	Y*a*	3.4548 (12)	3.2	1.294
Y*a*	Z*a*	3.5466 (13)	3.3	1.482
btc^3−^ (**1***b*)	X*b*	3.769 (11)	3.4	1.881
btc^3−^ (**1***b*)	Z*b*	3.694 (10)	3.2	1.87
*Xb*	Y*b*	3.416 (13)	3.4	0.195
Y*b*	Z*b*	3.544 (13)	3.5	0.347

**Table 6:** Hydrogen-bond geometry (Å, °)

	Graph-set descriptor	Type	*D*—H	H⋯*A*	*D*⋯*A*	*D*—H⋯*A*
N1*A*—H1*AA*⋯O1*A*^iv^	*D*(2)	*a*	0.883 (9)	1.730 (10)	2.6101 (17)	174.4 (18)
N2*A*—H2*A*⋯O4*A*^v^	*D*(2)	*b*	0.873 (9)	1.828 (10)	2.6888 (18)	168.5 (18)
N3*A*—H3*A*⋯O5*A*^vi^	*D*(2)	*c*	0.881 (9)	1.755 (10)	2.6309 (16)	172.3 (18)
N4*A*—H4*A*⋯O2*A*	*D*(2)	*d*	0.877 (9)	1.815 (10)	2.682 (2)	169.8 (18)
N5*A*—H5*AA*⋯O3*A*^i^	*D*(2)	*e*	0.868 (9)	1.749 (10)	2.6131 (17)	173.2 (19)
N6*A*—H6*A*⋯O6*A*^vii^	*D*(2)	*f*	0.873 (9)	1.857 (10)	2.713 (2)	166.7 (18)
N1*B*—H1*B*⋯O1*B*^i^	*D*(2)	*a*	0.88	1.64	2.510 (16)	169.2
N2*B*—H2*B*⋯O4*B*^vii^	*D*(2)	*b*	0.88	1.75	2.606 (16)	164.9
N3*B*—H3*B*⋯O5*B*^iii^	*D*(2)	*c*	0.88	1.66	2.522 (15)	167.6
N4*B*—H4*B*⋯O2*B*^i^	*D*(2)	*d*	0.88	1.75	2.598 (16)	161.8
N5*B*—H5*BA*⋯O3*B*^iv^	*D*(2)	*e*	0.88	1.65	2.518 (15)	168.4
N6*B*—H6*B*⋯O6*B*^v^	*D*(2)	*f*	0.88	1.83	2.648 (19)	153.2
C10*A*—H10*C*⋯O2*A*^iv^			0.98	2.42	3.390 (2)	170.8
C12*A*—H12*A*⋯O5*A*^v^			0.95	2.45	3.338 (2)	156
C14*A*—H14*B*⋯O6*A*^vi^			0.98	2.43	3.3867 (19)	165
C17*A*—H17*A*⋯O3*A*			0.95	2.44	3.3202 (19)	154.5
C18*A*—H18*A*⋯O1*A*			0.98	2.63	3.462 (2)	143.2
C18*A*—H18*C*⋯O4A^i^			0.98	2.45	3.371 (2)	156.5
C20*A*—H20*A*⋯O1*A*^vii^			0.95	2.38	3.2993 (19)	163.5
C10*B*—H10*D*⋯O2B^i^			0.98	2.61	3.51 (2)	151.4
C10*B*—H10*E*⋯O2B^ii^			0.98	2.05	2.85 (3)	136.8
C12*B*—H12*B*⋯O5*B*^vii^			0.95	2.58	3.475 (15)	157.1
C17*B*—H17*B*⋯O3*B*^i^			0.95	2.52	3.442 (16)	164.3

Symmetry codes: (i) 1 − *x*, 1 − *y*, 2 − *z*; (ii) 1 + *x*, *y*, *z*; (iii) −*x*, −*y*, 2 − *z*; (iv) 1 − *x*, 1 − *y*, 1 − *z*; (v) 1 − *x*, −*y*, 2 − *z*; (vi) −1 + *x*, 1 + *y*, *z*; (vii) 2 − *x*, 1 − *y*, 1 − *z*.

**Table 7:** Second-level graph sets in **1**

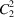 (12)	>*a*<*b*	*D*^2^_2_(9)	>*b*<*c*	*D*^2^_2_(9)	>*c*<*e*
*D*^2^_2_(9)	>*a*<*c*	*D*^2^_2_(9)	>*b*<*d*	*D*^2^_2_(5)	>*c*<*f*
*D*^2^_2_(5)	>*a*<*d*	*D*^2^_2_(5)	>*b*<*e*	*D*^2^_2_(9)	>*d*<*e*
*D*^2^_2_(9)	>*a*<*e*	*D*^2^_2_(9)	>*b*<*f*	*D*^2^_2_(9)	>*d*<*f*
*D*^2^_2_(9)	>*a*<*f*	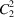 (12)	>*c*<*d*	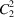 (12)	>*e*<*f*

**Table 8:** Inter­molecular inter­action contribution (%) from Hirshfeld surface analysis of **1**

	btc^3−^	btc^3−^	H2-mIm^+^	H2-mIm^+^	H2-mIm^+^	H2-mIm^+^	H2-mIm^+^	H2-mIm^+^
	**1** *a*	**1** *b*	X*a*	Y*a*	Z*a*	X*b*	Y*b*	Z*b*
O—O	0	1.3	–	–	–	–	–	–
O—C	2.2	2.8	–	–	–	–	–	–
O—H	56.2	52.8	–	–	–	–	–	–
O—N	0	0.1	–	–	–	–	–	–
C—O	2.7	2.9	0.3	0.2	0.5	0.6	0	0.8
C—C	5.3	6.9	5	2.7	5.2	6.5	4.7	6
C—H	12.2	9.8	3.7	5.1	3	0.4	0.5	0.9
C—N	2	2.4	1.1	2.3	1	2.3	4.2	2
H—N	1.3	1	1	2.3	1.3	0.6	1.1	0.6
H—H	15.2	17.5	50.2	55.4	49.3	54.6	62.9	53.4
H—C	1.4	0.1	7.5	3.6	7.1	3.9	1.1	5.1
H—O	1.4	2.4	24.5	21.8	26.1	24.6	19.2	24.7
N—O	–	–	0	0.1	0	0.1	0	0.1
N—C	–	–	2.8	2.1	2.7	4.3	4.2	3.9
N—H	–	–	3	2.6	2.8	1.8	1.2	2
N—N	–	–	0.9	2	1.1	0.4	1	0.6

**Table 9:** Experimental details

Crystal data	
Chemical formula	3C_4_H_7_N_2_^+^·C_9_H_3_O_6_^3−^
*M*_r_	456.46
Crystal system, space group	Triclinic, *P* 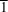
Temperature (K)	296
*a*, *b*, *c* (Å)	8.8634 (5), 10.3453 (7), 11.6777 (7)
α, β, γ (°)	74.199 (4), 79.749 (4), 87.580 (4)
*V* (Å^3^)	1013.85 (11)
*Z*	2
Radiation type	Mo *K*α
μ (mm^−1^)	0.11
Crystal size (mm)	0.1 × 0.08 × 0.07

Data collection	
Diffractometer	Bruker P4
Absorption correction	Multi-scan (*SADABS*; Krause *et al.*, 2015 ▸)
*T*_min_, *T*_max_	0.661, 0.746
No. of measured, independent and observed [*I* > 2σ(*I*)] reflections	18775, 4693, 3780
*R*_int_	0.033
(sin θ/λ)_max_ (Å^−1^)	0.652

Refinement	
*R*[*F*^2^ > 2σ(*F*^2^)], *wR*(*F*^2^), *S*	0.039, 0.102, 1.05
No. of reflections	4693
No. of parameters	584
No. of restraints	1806
H-atom treatment	H atoms treated by a mixture of independent and constrained refinement
Δρ_max_, Δρ_min_ (e Å^−3^)	0.36, −0.23

Computer programs: *APEX2* and *SAINT* (Bruker, 2016 ▸), *SHELXT2018/2* (Sheldrick, 2015*a* ▸), *SHELXL2018/3* (Sheldrick, 2015*b* ▸) and *OLEX2* 1.5 (Dolomanov *et al.*, 2009 ▸).

## supplementary crystallographic information

### Tris(2-methyl-1H-imidazol-3-ium) benzene-1,3,5-tricarboxylate Crystal data

**Table:**

3C_4_H_7_N_2_^+^·C_9_H_3_O_6_^3^^−^	*Z* = 2
*M**_r_* = 456.46	*F*(000) = 480
Triclinic, *P*1	*D*_x_ = 1.495 Mg m^−^^3^
*a* = 8.8634 (5) Å	Mo *K*α radiation, λ = 0.71073 Å
*b* = 10.3453 (7) Å	Cell parameters from 4963 reflections
*c* = 11.6777 (7) Å	θ = 2.7–27.6°
α = 74.199 (4)°	µ = 0.11 mm^−^^1^
β = 79.749 (4)°	*T* = 296 K
γ = 87.580 (4)°	Irregular, clear light colourless
*V* = 1013.85 (11) Å^3^	0.1 × 0.08 × 0.07 mm

### Tris(2-methyl-1H-imidazol-3-ium) benzene-1,3,5-tricarboxylate Data collection

**Table:**

Bruker P4 diffractometer	*R*_int_ = 0.033
Graphite monochromator	θ_max_ = 27.6°, θ_min_ = 1.8°
ω scans	*h* = −11→11
Absorption correction: multi-scan (SADABS; Krause *et al.*, 2015)	*k* = −13→13
*T*_min_ = 0.661, *T*_max_ = 0.746	*l* = −15→15
18775 measured reflections	Standard reflections: not measured; every not measured reflections
4693 independent reflections	intensity decay: not measured
3780 reflections with *I* > 2σ(*I*)	

### Tris(2-methyl-1H-imidazol-3-ium) benzene-1,3,5-tricarboxylate Refinement

**Table:**

Refinement on *F*^2^	Primary atom site location: dual
Least-squares matrix: full	Hydrogen site location: mixed
*R*[*F*^2^ > 2σ(*F*^2^)] = 0.039	H atoms treated by a mixture of independent and constrained refinement
*wR*(*F*^2^) = 0.102	*w* = 1/[σ^2^(*F*_o_^2^) + (0.0501*P*)^2^ + 0.3475*P*] where *P* = (*F*_o_^2^ + 2*F*_c_^2^)/3
*S* = 1.05	(Δ/σ)_max_ = 0.001
4693 reflections	Δρ_max_ = 0.36 e Å^−^^3^
584 parameters	Δρ_min_ = −0.23 e Å^−^^3^
1806 restraints	

### Tris(2-methyl-1H-imidazol-3-ium) benzene-1,3,5-tricarboxylate Special details

**Table:**

Geometry. All esds (except the esd in the dihedral angle between two l.s. planes) are estimated using the full covariance matrix. The cell esds are taken into account individually in the estimation of esds in distances, angles and torsion angles; correlations between esds in cell parameters are only used when they are defined by crystal symmetry. An approximate (isotropic) treatment of cell esds is used for estimating esds involving l.s. planes.

### Tris(2-methyl-1H-imidazol-3-ium) benzene-1,3,5-tricarboxylate Fractional atomic coordinates and isotropic or equivalent isotropic displacement parameters (Å^2^)

**Table:**

	*x*	*y*	*z*	*U*_iso_*/*U*_eq_	Occ. (<1)
O1A	0.63437 (13)	0.63491 (11)	0.46926 (9)	0.0164 (2)	0.9099 (9)
O2A	0.43997 (12)	0.69982 (11)	0.59090 (10)	0.0191 (3)	0.9099 (9)
O3A	0.29536 (12)	0.41347 (12)	1.00672 (10)	0.0186 (3)	0.9099 (9)
O4A	0.44291 (12)	0.24354 (10)	1.08539 (9)	0.0176 (2)	0.9099 (9)
O5A	0.88019 (13)	0.11476 (11)	0.80957 (10)	0.0217 (3)	0.9099 (9)
O6A	0.92786 (12)	0.22264 (11)	0.61337 (9)	0.0190 (2)	0.9099 (9)
C1A	0.54608 (16)	0.62073 (15)	0.56953 (14)	0.0130 (3)	0.9099 (9)
C2A	0.41160 (18)	0.33842 (14)	1.00035 (14)	0.0127 (3)	0.9099 (9)
C3A	0.85593 (17)	0.20775 (15)	0.71862 (13)	0.0137 (3)	0.9099 (9)
C4A	0.57684 (18)	0.50083 (14)	0.67027 (14)	0.0118 (3)	0.9099 (9)
C5A	0.48727 (17)	0.47639 (15)	0.78478 (14)	0.0119 (3)	0.9099 (9)
H5A	0.405067	0.535044	0.799311	0.014*	0.9099 (9)
C6A	0.51636 (17)	0.36719 (16)	0.87845 (13)	0.0114 (3)	0.9099 (9)
C7A	0.63867 (18)	0.28287 (15)	0.85651 (14)	0.0115 (3)	0.9099 (9)
H7A	0.661151	0.209755	0.920485	0.014*	0.9099 (9)
C8A	0.72825 (16)	0.30428 (15)	0.74229 (14)	0.0117 (3)	0.9099 (9)
C9A	0.69628 (17)	0.41354 (15)	0.64951 (13)	0.0120 (3)	0.9099 (9)
H9A	0.756694	0.428611	0.571195	0.014*	0.9099 (9)
N1A	0.36212 (15)	0.17449 (12)	0.73067 (12)	0.0148 (3)	0.9099 (9)
H1A	0.367 (2)	0.2355 (15)	0.6605 (11)	0.022*	0.9099 (9)
N2A	0.4159 (3)	−0.0048 (2)	0.86170 (16)	0.0140 (4)	0.9099 (9)
H2A	0.4619 (19)	−0.0805 (12)	0.8884 (16)	0.021*	0.9099 (9)
C10A	0.5638 (2)	0.03092 (17)	0.65126 (16)	0.0202 (4)	0.9099 (9)
H10A	0.534175	−0.053945	0.639115	0.030*	0.9099 (9)
H10B	0.664662	0.021176	0.675921	0.030*	0.9099 (9)
H10C	0.568484	0.102221	0.575648	0.030*	0.9099 (9)
C11A	0.44916 (16)	0.06610 (14)	0.74657 (13)	0.0135 (3)	0.9099 (9)
C12A	0.30468 (17)	0.06181 (15)	0.92221 (14)	0.0162 (3)	0.9099 (9)
H12A	0.260489	0.034459	1.005449	0.019*	0.9099 (9)
C13A	0.27126 (19)	0.17348 (17)	0.83954 (15)	0.0167 (3)	0.9099 (9)
H13A	0.198107	0.239584	0.853903	0.020*	0.9099 (9)
N3A	0.06717 (14)	0.90954 (13)	0.81578 (12)	0.0147 (3)	0.9099 (9)
H3A	0.0060 (18)	0.9796 (14)	0.8066 (17)	0.022*	0.9099 (9)
N4A	0.2353 (4)	0.7711 (3)	0.7613 (2)	0.0136 (4)	0.9099 (9)
H4A	0.3034 (17)	0.7388 (18)	0.7122 (14)	0.020*	0.9099 (9)
C14A	0.1663 (2)	0.96187 (19)	0.59451 (15)	0.0167 (4)	0.9099 (9)
H14A	0.268061	1.003764	0.565408	0.025*	0.9099 (9)
H14B	0.087809	1.031841	0.589348	0.025*	0.9099 (9)
H14C	0.149788	0.903210	0.544569	0.025*	0.9099 (9)
C15A	0.15618 (17)	0.88171 (15)	0.72134 (13)	0.0130 (3)	0.9099 (9)
C16A	0.09140 (19)	0.81487 (18)	0.91902 (16)	0.0165 (4)	0.9099 (9)
H16A	0.043111	0.811425	0.999237	0.020*	0.9099 (9)
C17A	0.19652 (17)	0.72754 (15)	0.88554 (13)	0.0151 (3)	0.9099 (9)
H17A	0.235948	0.651262	0.937315	0.018*	0.9099 (9)
N5A	0.90654 (15)	0.58146 (13)	0.80199 (12)	0.0159 (3)	0.9099 (9)
H5AA	0.8356 (17)	0.5879 (19)	0.8618 (13)	0.024*	0.9099 (9)
N6A	1.0223 (4)	0.6183 (3)	0.61691 (17)	0.0158 (5)	0.9099 (9)
H6A	1.047 (2)	0.6590 (17)	0.5401 (9)	0.024*	0.9099 (9)
C18A	0.8036 (2)	0.77104 (17)	0.65572 (15)	0.0173 (4)	0.9099 (9)
H18A	0.775661	0.772415	0.577879	0.026*	0.9099 (9)
H18B	0.854750	0.855706	0.648619	0.026*	0.9099 (9)
H18C	0.710800	0.760067	0.717405	0.026*	0.9099 (9)
C19A	0.90804 (17)	0.65821 (15)	0.69068 (14)	0.0140 (3)	0.9099 (9)
C20A	1.09638 (18)	0.51212 (15)	0.68442 (14)	0.0183 (3)	0.9099 (9)
H20A	1.181907	0.464382	0.655269	0.022*	0.9099 (9)
C21A	1.0234 (2)	0.48953 (18)	0.79978 (15)	0.0186 (4)	0.9099 (9)
H21A	1.048088	0.422387	0.867299	0.022*	0.9099 (9)
O1B	0.3361 (14)	0.3467 (11)	1.0512 (9)	0.021 (2)	0.0901 (9)
O2B	0.2768 (12)	0.5341 (10)	0.9276 (9)	0.019 (2)	0.0901 (9)
O3B	0.5623 (13)	0.6743 (11)	0.5010 (11)	0.019 (2)	0.0901 (9)
O4B	0.7474 (12)	0.5493 (10)	0.4393 (8)	0.0185 (19)	0.0901 (9)
O5B	0.9052 (12)	0.1340 (12)	0.7314 (10)	0.022 (2)	0.0901 (9)
O6B	0.7760 (13)	0.0691 (10)	0.9168 (8)	0.0217 (3)	0.0901 (9)
C1B	0.3620 (15)	0.4349 (12)	0.9506 (10)	0.0154 (17)	0.0901 (9)
C2B	0.6491 (15)	0.5726 (12)	0.5215 (9)	0.0155 (17)	0.0901 (9)
C3B	0.8013 (15)	0.1528 (11)	0.8159 (9)	0.0155 (15)	0.0901 (9)
C4B	0.4914 (16)	0.4160 (15)	0.8519 (11)	0.0139 (15)	0.0901 (9)
C5B	0.5144 (17)	0.4987 (15)	0.7351 (10)	0.0130 (15)	0.0901 (9)
H5B	0.450408	0.574762	0.715534	0.016*	0.0901 (9)
C6B	0.6278 (17)	0.4738 (13)	0.6461 (9)	0.0118 (3)	0.0901 (9)
C7B	0.7185 (17)	0.3616 (13)	0.6748 (11)	0.0120 (3)	0.0901 (9)
H7B	0.795895	0.343054	0.613952	0.014*	0.0901 (9)
C8B	0.6991 (17)	0.2759 (13)	0.7898 (11)	0.0143 (14)	0.0901 (9)
C9B	0.5855 (17)	0.3057 (15)	0.8771 (12)	0.0140 (15)	0.0901 (9)
H9B	0.571941	0.248118	0.956982	0.017*	0.0901 (9)
N1B	0.8512 (14)	0.6448 (12)	0.7625 (10)	0.0162 (15)	0.0901 (9)
H1B	0.794429	0.643147	0.832866	0.019*	0.0901 (9)
N2B	1.022 (4)	0.596 (3)	0.6250 (14)	0.0151 (19)	0.0901 (9)
H2B	1.099861	0.555833	0.590398	0.018*	0.0901 (9)
C10B	1.027 (3)	0.459 (2)	0.8362 (16)	0.0186 (4)	0.0901 (9)
H10D	0.973669	0.462481	0.916474	0.028*	0.0901 (9)
H10E	1.136675	0.473684	0.829447	0.028*	0.0901 (9)
H10F	1.009621	0.370858	0.824488	0.028*	0.0901 (9)
C11B	0.9660 (16)	0.5645 (13)	0.7432 (11)	0.0172 (16)	0.0901 (9)
C12B	0.9386 (16)	0.7024 (13)	0.5652 (10)	0.0168 (18)	0.0901 (9)
H12B	0.951250	0.744914	0.481027	0.020*	0.0901 (9)
C13B	0.8361 (19)	0.7308 (16)	0.6542 (11)	0.0144 (18)	0.0901 (9)
H13B	0.763155	0.801145	0.643027	0.017*	0.0901 (9)
N3B	0.9101 (15)	0.0588 (12)	1.2644 (10)	0.020 (2)	0.0901 (9)
H3B	0.972182	−0.007821	1.255590	0.024*	0.0901 (9)
N4B	0.780 (5)	0.241 (3)	1.224 (2)	0.020 (3)	0.0901 (9)
H4B	0.741883	0.315890	1.182443	0.024*	0.0901 (9)
C14B	0.967 (2)	0.2003 (19)	1.0515 (11)	0.019 (3)	0.0901 (9)
H14D	0.990040	0.116708	1.028014	0.029*	0.0901 (9)
H14E	1.061878	0.250271	1.041044	0.029*	0.0901 (9)
H14F	0.897947	0.255024	1.000667	0.029*	0.0901 (9)
C15B	0.8916 (17)	0.1686 (14)	1.1790 (10)	0.019 (2)	0.0901 (9)
C16B	0.817 (2)	0.0660 (16)	1.3684 (11)	0.017 (3)	0.0901 (9)
H16B	0.811200	0.001269	1.444487	0.020*	0.0901 (9)
C17B	0.7345 (17)	0.1797 (14)	1.3463 (12)	0.021 (2)	0.0901 (9)
H17B	0.661054	0.210989	1.402322	0.025*	0.0901 (9)
N5B	0.4442 (13)	0.1450 (11)	0.6925 (9)	0.0148 (3)	0.0901 (9)
H5BA	0.437910	0.215239	0.631013	0.018*	0.0901 (9)
N6B	0.410 (3)	0.013 (3)	0.8728 (14)	0.016 (2)	0.0901 (9)
H6B	0.376045	−0.018373	0.950791	0.019*	0.0901 (9)
C18B	0.2189 (18)	0.1957 (19)	0.8343 (17)	0.0167 (3)	0.0901 (9)
H18D	0.236520	0.290934	0.791949	0.025*	0.0901 (9)
H18E	0.204232	0.184000	0.921700	0.025*	0.0901 (9)
H18F	0.127233	0.164192	0.813587	0.025*	0.0901 (9)
C19B	0.3520 (14)	0.1176 (14)	0.7978 (11)	0.0169 (16)	0.0901 (9)
C20B	0.5330 (16)	−0.0392 (13)	0.8061 (11)	0.0171 (18)	0.0901 (9)
H20B	0.590742	−0.117429	0.833557	0.021*	0.0901 (9)
C21B	0.5516 (18)	0.0457 (15)	0.6950 (11)	0.0161 (18)	0.0901 (9)
H21B	0.627299	0.038191	0.628490	0.019*	0.0901 (9)

### Tris(2-methyl-1H-imidazol-3-ium) benzene-1,3,5-tricarboxylate Atomic displacement parameters (Å^2^)

**Table:**

	*U*^11^	*U*^22^	*U*^33^	*U*^12^	*U*^13^	*U*^23^
O1A	0.0192 (6)	0.0154 (6)	0.0093 (5)	0.0061 (5)	0.0031 (4)	0.0011 (5)
O2A	0.0218 (6)	0.0166 (5)	0.0133 (5)	0.0117 (4)	0.0018 (4)	0.0003 (4)
O3A	0.0199 (6)	0.0180 (6)	0.0116 (6)	0.0103 (5)	0.0034 (5)	0.0007 (5)
O4A	0.0213 (5)	0.0157 (5)	0.0098 (5)	0.0077 (4)	0.0022 (4)	0.0025 (4)
O5A	0.0259 (6)	0.0188 (6)	0.0132 (6)	0.0139 (5)	0.0021 (5)	0.0020 (5)
O6A	0.0214 (6)	0.0192 (5)	0.0115 (5)	0.0094 (4)	0.0033 (4)	−0.0011 (4)
C1A	0.0153 (7)	0.0112 (7)	0.0107 (8)	0.0028 (6)	−0.0008 (6)	−0.0015 (6)
C2A	0.0148 (7)	0.0111 (7)	0.0110 (7)	0.0030 (6)	−0.0009 (6)	−0.0023 (6)
C3A	0.0163 (7)	0.0117 (7)	0.0113 (7)	0.0037 (6)	−0.0015 (6)	−0.0012 (6)
C4A	0.0139 (8)	0.0098 (7)	0.0097 (7)	0.0027 (5)	−0.0005 (6)	−0.0009 (5)
C5A	0.0128 (7)	0.0110 (7)	0.0102 (8)	0.0041 (6)	−0.0001 (6)	−0.0020 (6)
C6A	0.0130 (7)	0.0102 (7)	0.0100 (7)	0.0019 (6)	−0.0001 (6)	−0.0024 (6)
C7A	0.0145 (8)	0.0085 (7)	0.0098 (7)	0.0029 (6)	−0.0020 (6)	0.0000 (5)
C8A	0.0119 (7)	0.0109 (7)	0.0111 (8)	0.0029 (6)	−0.0001 (6)	−0.0024 (6)
C9A	0.0140 (7)	0.0113 (7)	0.0081 (7)	0.0030 (6)	0.0006 (5)	−0.0005 (6)
N1A	0.0186 (6)	0.0116 (6)	0.0115 (6)	0.0030 (5)	−0.0014 (5)	0.0002 (5)
N2A	0.0175 (7)	0.0101 (9)	0.0123 (7)	0.0037 (6)	−0.0013 (6)	−0.0008 (6)
C10A	0.0218 (8)	0.0188 (8)	0.0145 (8)	0.0073 (6)	0.0036 (7)	−0.0006 (7)
C11A	0.0152 (7)	0.0114 (7)	0.0121 (7)	0.0019 (5)	−0.0016 (6)	−0.0007 (6)
C12A	0.0185 (7)	0.0139 (7)	0.0146 (7)	0.0016 (6)	0.0004 (6)	−0.0034 (6)
C13A	0.0185 (9)	0.0135 (8)	0.0149 (7)	0.0041 (6)	0.0020 (7)	−0.0024 (6)
N3A	0.0150 (6)	0.0129 (6)	0.0149 (7)	0.0057 (5)	−0.0006 (5)	−0.0036 (5)
N4A	0.0144 (10)	0.0119 (9)	0.0128 (9)	0.0046 (8)	0.0003 (7)	−0.0028 (6)
C14A	0.0193 (8)	0.0161 (9)	0.0114 (8)	0.0048 (6)	−0.0003 (7)	−0.0003 (7)
C15A	0.0128 (7)	0.0117 (7)	0.0140 (8)	0.0020 (6)	−0.0019 (6)	−0.0031 (6)
C16A	0.0174 (8)	0.0161 (8)	0.0135 (8)	0.0026 (7)	0.0006 (6)	−0.0024 (7)
C17A	0.0180 (7)	0.0137 (7)	0.0121 (7)	0.0027 (6)	−0.0022 (6)	−0.0014 (6)
N5A	0.0177 (7)	0.0157 (6)	0.0110 (6)	0.0033 (5)	0.0037 (5)	−0.0023 (5)
N6A	0.0184 (7)	0.0145 (13)	0.0105 (7)	0.0027 (8)	0.0032 (6)	−0.0008 (6)
C18A	0.0162 (8)	0.0148 (8)	0.0176 (8)	0.0039 (6)	−0.0001 (6)	−0.0010 (6)
C19A	0.0146 (7)	0.0130 (7)	0.0131 (7)	−0.0002 (6)	0.0009 (6)	−0.0036 (6)
C20A	0.0202 (8)	0.0145 (7)	0.0165 (8)	0.0056 (6)	0.0015 (6)	−0.0019 (6)
C21A	0.0225 (7)	0.0150 (9)	0.0137 (9)	0.0063 (7)	0.0004 (8)	0.0005 (7)
O1B	0.021 (4)	0.017 (4)	0.016 (4)	0.009 (4)	0.006 (4)	0.000 (3)
O2B	0.024 (4)	0.020 (4)	0.009 (4)	0.009 (3)	0.002 (3)	−0.002 (3)
O3B	0.022 (4)	0.019 (4)	0.007 (4)	0.004 (3)	0.003 (4)	0.005 (3)
O4B	0.025 (4)	0.016 (4)	0.007 (3)	0.004 (3)	0.002 (3)	0.004 (3)
O5B	0.027 (4)	0.014 (4)	0.015 (4)	0.010 (3)	0.001 (3)	0.007 (3)
O6B	0.0259 (6)	0.0188 (6)	0.0132 (6)	0.0139 (5)	0.0021 (5)	0.0020 (5)
C1B	0.018 (3)	0.014 (3)	0.012 (3)	0.003 (3)	0.001 (3)	−0.001 (3)
C2B	0.019 (3)	0.014 (3)	0.010 (3)	0.003 (3)	0.001 (2)	0.001 (2)
C3B	0.018 (3)	0.013 (2)	0.012 (2)	0.005 (2)	−0.001 (2)	0.001 (2)
C4B	0.017 (2)	0.012 (2)	0.009 (2)	0.006 (2)	0.001 (2)	0.001 (2)
C5B	0.017 (2)	0.011 (2)	0.008 (2)	0.003 (2)	0.000 (2)	0.001 (2)
C6B	0.0139 (8)	0.0098 (7)	0.0097 (7)	0.0027 (5)	−0.0005 (6)	−0.0009 (5)
C7B	0.0140 (7)	0.0113 (7)	0.0081 (7)	0.0030 (6)	0.0006 (5)	−0.0005 (6)
C8B	0.018 (2)	0.012 (2)	0.009 (2)	0.005 (2)	0.001 (2)	0.001 (2)
C9B	0.017 (2)	0.013 (2)	0.009 (2)	0.005 (2)	0.001 (2)	−0.001 (2)
N1B	0.020 (3)	0.015 (3)	0.009 (3)	0.003 (2)	0.001 (2)	0.002 (2)
N2B	0.019 (3)	0.013 (3)	0.010 (3)	0.003 (3)	0.003 (3)	−0.002 (3)
C10B	0.0225 (7)	0.0150 (9)	0.0137 (9)	0.0063 (7)	0.0004 (8)	0.0005 (7)
C11B	0.019 (3)	0.015 (3)	0.012 (3)	0.003 (2)	0.003 (2)	0.001 (2)
C12B	0.020 (3)	0.013 (3)	0.012 (3)	0.005 (3)	0.001 (3)	0.001 (3)
C13B	0.018 (3)	0.012 (3)	0.009 (3)	0.002 (3)	0.002 (3)	0.001 (3)
N3B	0.027 (5)	0.019 (4)	0.013 (4)	0.007 (4)	0.005 (4)	−0.009 (3)
N4B	0.023 (5)	0.015 (4)	0.018 (4)	0.009 (4)	0.002 (4)	−0.003 (4)
C14B	0.025 (6)	0.014 (5)	0.014 (4)	0.008 (5)	0.001 (4)	0.001 (4)
C15B	0.022 (4)	0.016 (4)	0.015 (4)	0.006 (3)	0.002 (3)	−0.006 (3)
C16B	0.021 (5)	0.016 (5)	0.013 (5)	0.008 (4)	0.000 (4)	−0.006 (4)
C17B	0.024 (5)	0.018 (4)	0.015 (4)	0.011 (4)	0.003 (4)	−0.001 (4)
N5B	0.0186 (6)	0.0116 (6)	0.0115 (6)	0.0030 (5)	−0.0014 (5)	0.0002 (5)
N6B	0.020 (3)	0.013 (3)	0.010 (3)	0.003 (3)	−0.001 (3)	0.003 (3)
C18B	0.0185 (9)	0.0135 (8)	0.0149 (7)	0.0041 (6)	0.0020 (7)	−0.0024 (6)
C19B	0.019 (3)	0.013 (3)	0.013 (3)	0.004 (2)	0.000 (2)	0.003 (2)
C20B	0.020 (3)	0.015 (3)	0.012 (3)	0.004 (3)	0.002 (3)	0.002 (3)
C21B	0.021 (3)	0.012 (3)	0.011 (3)	0.004 (3)	0.002 (3)	0.001 (3)

### Tris(2-methyl-1H-imidazol-3-ium) benzene-1,3,5-tricarboxylate Geometric parameters (Å, º)

**Table:**

O1A—C1A	1.2629 (18)	O1B—C1B	1.266 (9)
O2A—C1A	1.2565 (17)	O2B—C1B	1.246 (9)
O3A—C2A	1.2674 (17)	O3B—C2B	1.271 (9)
O4A—C2A	1.2534 (17)	O4B—C2B	1.245 (9)
O5A—C3A	1.2677 (18)	O5B—C3B	1.275 (9)
O6A—C3A	1.2519 (18)	O6B—C3B	1.247 (9)
C1A—C4A	1.513 (2)	C1B—C4B	1.522 (9)
C2A—C6A	1.516 (2)	C2B—C6B	1.518 (9)
C3A—C8A	1.5159 (19)	C3B—C8B	1.524 (8)
C4A—C5A	1.392 (2)	C4B—C5B	1.384 (9)
C4A—C9A	1.3936 (19)	C4B—C9B	1.381 (9)
C5A—H5A	0.9500	C5B—H5B	0.9500
C5A—C6A	1.394 (2)	C5B—C6B	1.382 (9)
C6A—C7A	1.395 (2)	C6B—C7B	1.383 (9)
C7A—H7A	0.9500	C7B—H7B	0.9500
C7A—C8A	1.391 (2)	C7B—C8B	1.379 (9)
C8A—C9A	1.395 (2)	C8B—C9B	1.388 (9)
C9A—H9A	0.9500	C9B—H9B	0.9500
N1A—H1A	0.883 (9)	N1B—H1B	0.8800
N1A—C11A	1.3234 (18)	N1B—C11B	1.313 (9)
N1A—C13A	1.376 (2)	N1B—C13B	1.360 (9)
N2A—H2A	0.873 (9)	N2B—H2B	0.8800
N2A—C11A	1.331 (2)	N2B—C11B	1.336 (10)
N2A—C12A	1.381 (2)	N2B—C12B	1.397 (10)
C10A—H10A	0.9800	C10B—H10D	0.9800
C10A—H10B	0.9800	C10B—H10E	0.9800
C10A—H10C	0.9800	C10B—H10F	0.9800
C10A—C11A	1.480 (2)	C10B—C11B	1.473 (9)
C12A—H12A	0.9500	C12B—H12B	0.9500
C12A—C13A	1.349 (2)	C12B—C13B	1.341 (9)
C13A—H13A	0.9500	C13B—H13B	0.9500
N3A—H3A	0.881 (9)	N3B—H3B	0.8800
N3A—C15A	1.3287 (19)	N3B—C15B	1.318 (9)
N3A—C16A	1.375 (2)	N3B—C16B	1.360 (9)
N4A—H4A	0.877 (9)	N4B—H4B	0.8800
N4A—C15A	1.333 (2)	N4B—C15B	1.334 (9)
N4A—C17A	1.381 (3)	N4B—C17B	1.394 (10)
C14A—H14A	0.9800	C14B—H14D	0.9800
C14A—H14B	0.9800	C14B—H14E	0.9800
C14A—H14C	0.9800	C14B—H14F	0.9800
C14A—C15A	1.476 (2)	C14B—C15B	1.472 (9)
C16A—H16A	0.9500	C16B—H16B	0.9500
C16A—C17A	1.352 (2)	C16B—C17B	1.347 (10)
C17A—H17A	0.9500	C17B—H17B	0.9500
N5A—H5AA	0.868 (9)	N5B—H5BA	0.8800
N5A—C19A	1.324 (2)	N5B—C19B	1.316 (9)
N5A—C21A	1.378 (2)	N5B—C21B	1.368 (9)
N6A—H6A	0.873 (9)	N6B—H6B	0.8800
N6A—C19A	1.337 (2)	N6B—C19B	1.343 (10)
N6A—C20A	1.383 (2)	N6B—C20B	1.400 (10)
C18A—H18A	0.9800	C18B—H18D	0.9800
C18A—H18B	0.9800	C18B—H18E	0.9800
C18A—H18C	0.9800	C18B—H18F	0.9800
C18A—C19A	1.473 (2)	C18B—C19B	1.468 (9)
C20A—H20A	0.9500	C20B—H20B	0.9500
C20A—C21A	1.349 (2)	C20B—C21B	1.341 (10)
C21A—H21A	0.9500	C21B—H21B	0.9500
			
O1A—C1A—C4A	115.95 (13)	O1B—C1B—C4B	120.2 (9)
O2A—C1A—O1A	124.87 (15)	O2B—C1B—O1B	121.0 (10)
O2A—C1A—C4A	119.16 (14)	O2B—C1B—C4B	118.6 (9)
O3A—C2A—C6A	115.77 (13)	O3B—C2B—C6B	119.9 (9)
O4A—C2A—O3A	124.98 (15)	O4B—C2B—O3B	120.8 (10)
O4A—C2A—C6A	119.24 (14)	O4B—C2B—C6B	119.2 (9)
O5A—C3A—C8A	116.00 (13)	O5B—C3B—C8B	119.0 (8)
O6A—C3A—O5A	124.60 (14)	O6B—C3B—O5B	121.5 (10)
O6A—C3A—C8A	119.40 (13)	O6B—C3B—C8B	119.4 (9)
C5A—C4A—C1A	120.71 (14)	C5B—C4B—C1B	123.5 (9)
C5A—C4A—C9A	119.10 (13)	C9B—C4B—C1B	118.8 (9)
C9A—C4A—C1A	120.20 (14)	C9B—C4B—C5B	117.7 (9)
C4A—C5A—H5A	119.5	C4B—C5B—H5B	119.1
C4A—C5A—C6A	121.00 (13)	C6B—C5B—C4B	121.8 (9)
C6A—C5A—H5A	119.5	C6B—C5B—H5B	119.1
C5A—C6A—C2A	119.89 (14)	C5B—C6B—C2B	118.4 (9)
C5A—C6A—C7A	118.99 (14)	C5B—C6B—C7B	118.7 (9)
C7A—C6A—C2A	121.08 (14)	C7B—C6B—C2B	122.9 (9)
C6A—C7A—H7A	119.5	C6B—C7B—H7B	119.2
C8A—C7A—C6A	120.96 (13)	C8B—C7B—C6B	121.5 (9)
C8A—C7A—H7A	119.5	C8B—C7B—H7B	119.2
C7A—C8A—C3A	120.22 (13)	C7B—C8B—C3B	119.2 (9)
C7A—C8A—C9A	119.10 (13)	C7B—C8B—C9B	118.0 (9)
C9A—C8A—C3A	120.67 (14)	C9B—C8B—C3B	122.8 (9)
C4A—C9A—C8A	120.84 (13)	C4B—C9B—C8B	122.3 (9)
C4A—C9A—H9A	119.6	C4B—C9B—H9B	118.8
C8A—C9A—H9A	119.6	C8B—C9B—H9B	118.8
C11A—N1A—H1A	123.1 (12)	C11B—N1B—H1B	126.2
C11A—N1A—C13A	108.45 (13)	C11B—N1B—C13B	107.6 (9)
C13A—N1A—H1A	128.4 (12)	C13B—N1B—H1B	126.2
C11A—N2A—H2A	121.3 (13)	C11B—N2B—H2B	125.5
C11A—N2A—C12A	108.88 (14)	C11B—N2B—C12B	109.0 (9)
C12A—N2A—H2A	129.8 (13)	C12B—N2B—H2B	125.5
H10A—C10A—H10B	109.5	H10D—C10B—H10E	109.5
H10A—C10A—H10C	109.5	H10D—C10B—H10F	109.5
H10B—C10A—H10C	109.5	H10E—C10B—H10F	109.5
C11A—C10A—H10A	109.5	C11B—C10B—H10D	109.5
C11A—C10A—H10B	109.5	C11B—C10B—H10E	109.5
C11A—C10A—H10C	109.5	C11B—C10B—H10F	109.5
N1A—C11A—N2A	108.61 (14)	N1B—C11B—N2B	109.0 (8)
N1A—C11A—C10A	125.15 (14)	N1B—C11B—C10B	126.0 (11)
N2A—C11A—C10A	126.24 (14)	N2B—C11B—C10B	125.0 (11)
N2A—C12A—H12A	126.8	N2B—C12B—H12B	128.0
C13A—C12A—N2A	106.30 (15)	C13B—C12B—N2B	104.0 (9)
C13A—C12A—H12A	126.8	C13B—C12B—H12B	128.0
N1A—C13A—H13A	126.1	N1B—C13B—H13B	124.8
C12A—C13A—N1A	107.75 (14)	C12B—C13B—N1B	110.4 (9)
C12A—C13A—H13A	126.1	C12B—C13B—H13B	124.8
C15A—N3A—H3A	121.1 (12)	C15B—N3B—H3B	125.8
C15A—N3A—C16A	108.74 (13)	C15B—N3B—C16B	108.3 (9)
C16A—N3A—H3A	130.1 (12)	C16B—N3B—H3B	125.8
C15A—N4A—H4A	121.7 (13)	C15B—N4B—H4B	125.7
C15A—N4A—C17A	108.96 (15)	C15B—N4B—C17B	108.6 (9)
C17A—N4A—H4A	129.2 (13)	C17B—N4B—H4B	125.7
H14A—C14A—H14B	109.5	H14D—C14B—H14E	109.5
H14A—C14A—H14C	109.5	H14D—C14B—H14F	109.5
H14B—C14A—H14C	109.5	H14E—C14B—H14F	109.5
C15A—C14A—H14A	109.5	C15B—C14B—H14D	109.5
C15A—C14A—H14B	109.5	C15B—C14B—H14E	109.5
C15A—C14A—H14C	109.5	C15B—C14B—H14F	109.5
N3A—C15A—N4A	108.31 (15)	N3B—C15B—N4B	108.7 (9)
N3A—C15A—C14A	125.30 (14)	N3B—C15B—C14B	126.4 (11)
N4A—C15A—C14A	126.38 (16)	N4B—C15B—C14B	124.5 (11)
N3A—C16A—H16A	126.2	N3B—C16B—H16B	125.5
C17A—C16A—N3A	107.51 (15)	C17B—C16B—N3B	109.1 (9)
C17A—C16A—H16A	126.2	C17B—C16B—H16B	125.5
N4A—C17A—H17A	126.8	N4B—C17B—H17B	127.4
C16A—C17A—N4A	106.47 (14)	C16B—C17B—N4B	105.1 (9)
C16A—C17A—H17A	126.8	C16B—C17B—H17B	127.4
C19A—N5A—H5AA	122.0 (13)	C19B—N5B—H5BA	125.8
C19A—N5A—C21A	108.61 (13)	C19B—N5B—C21B	108.4 (8)
C21A—N5A—H5AA	129.1 (13)	C21B—N5B—H5BA	125.8
C19A—N6A—H6A	123.4 (13)	C19B—N6B—H6B	125.7
C19A—N6A—C20A	108.66 (15)	C19B—N6B—C20B	108.6 (9)
C20A—N6A—H6A	127.8 (13)	C20B—N6B—H6B	125.7
H18A—C18A—H18B	109.5	H18D—C18B—H18E	109.5
H18A—C18A—H18C	109.5	H18D—C18B—H18F	109.5
H18B—C18A—H18C	109.5	H18E—C18B—H18F	109.5
C19A—C18A—H18A	109.5	C19B—C18B—H18D	109.5
C19A—C18A—H18B	109.5	C19B—C18B—H18E	109.5
C19A—C18A—H18C	109.5	C19B—C18B—H18F	109.5
N5A—C19A—N6A	108.55 (14)	N5B—C19B—N6B	108.2 (9)
N5A—C19A—C18A	125.28 (14)	N5B—C19B—C18B	127.0 (11)
N6A—C19A—C18A	126.14 (15)	N6B—C19B—C18B	124.5 (11)
N6A—C20A—H20A	126.7	N6B—C20B—H20B	127.5
C21A—C20A—N6A	106.55 (14)	C21B—C20B—N6B	105.0 (9)
C21A—C20A—H20A	126.7	C21B—C20B—H20B	127.5
N5A—C21A—H21A	126.2	N5B—C21B—H21B	125.4
C20A—C21A—N5A	107.63 (13)	C20B—C21B—N5B	109.2 (9)
C20A—C21A—H21A	126.2	C20B—C21B—H21B	125.4
			
O1A—C1A—C4A—C5A	179.42 (14)	O1B—C1B—C4B—C5B	171.5 (18)
O1A—C1A—C4A—C9A	−0.3 (2)	O1B—C1B—C4B—C9B	−4 (3)
O2A—C1A—C4A—C5A	1.2 (2)	O2B—C1B—C4B—C5B	−4 (3)
O2A—C1A—C4A—C9A	−178.44 (14)	O2B—C1B—C4B—C9B	−179.6 (17)
O3A—C2A—C6A—C5A	4.3 (2)	O3B—C2B—C6B—C5B	−1 (3)
O3A—C2A—C6A—C7A	−173.16 (14)	O3B—C2B—C6B—C7B	−179.7 (16)
O4A—C2A—C6A—C5A	−176.84 (14)	O4B—C2B—C6B—C5B	−177.6 (16)
O4A—C2A—C6A—C7A	5.7 (2)	O4B—C2B—C6B—C7B	4 (3)
O5A—C3A—C8A—C7A	−4.4 (2)	O5B—C3B—C8B—C7B	2 (3)
O5A—C3A—C8A—C9A	177.30 (14)	O5B—C3B—C8B—C9B	−178.6 (18)
O6A—C3A—C8A—C7A	174.65 (14)	O6B—C3B—C8B—C7B	−174.1 (17)
O6A—C3A—C8A—C9A	−3.7 (2)	O6B—C3B—C8B—C9B	5 (3)
C1A—C4A—C5A—C6A	−179.09 (14)	C1B—C4B—C5B—C6B	−176.2 (17)
C1A—C4A—C9A—C8A	178.51 (14)	C1B—C4B—C9B—C8B	175.4 (17)
C2A—C6A—C7A—C8A	175.60 (14)	C2B—C6B—C7B—C8B	178.0 (17)
C3A—C8A—C9A—C4A	178.58 (14)	C3B—C8B—C9B—C4B	−178.6 (17)
C4A—C5A—C6A—C2A	−176.59 (14)	C4B—C5B—C6B—C2B	−177.7 (17)
C4A—C5A—C6A—C7A	0.9 (2)	C4B—C5B—C6B—C7B	1 (3)
C5A—C4A—C9A—C8A	−1.2 (2)	C5B—C4B—C9B—C8B	−1 (3)
C5A—C6A—C7A—C8A	−1.8 (2)	C5B—C6B—C7B—C8B	−1 (3)
C6A—C7A—C8A—C3A	−177.06 (14)	C6B—C7B—C8B—C3B	179.3 (16)
C6A—C7A—C8A—C9A	1.3 (2)	C6B—C7B—C8B—C9B	0 (3)
C7A—C8A—C9A—C4A	0.2 (2)	C7B—C8B—C9B—C4B	1 (3)
C9A—C4A—C5A—C6A	0.6 (2)	C9B—C4B—C5B—C6B	−1 (3)
N2A—C12A—C13A—N1A	−0.4 (2)	N2B—C12B—C13B—N1B	−2 (3)
C11A—N1A—C13A—C12A	−0.12 (19)	C11B—N1B—C13B—C12B	2 (2)
C11A—N2A—C12A—C13A	0.8 (3)	C11B—N2B—C12B—C13B	1 (4)
C12A—N2A—C11A—N1A	−0.8 (3)	C12B—N2B—C11B—N1B	0 (4)
C12A—N2A—C11A—C10A	179.21 (17)	C12B—N2B—C11B—C10B	−179 (2)
C13A—N1A—C11A—N2A	0.6 (2)	C13B—N1B—C11B—N2B	−1 (3)
C13A—N1A—C11A—C10A	−179.45 (16)	C13B—N1B—C11B—C10B	177 (2)
N3A—C16A—C17A—N4A	0.2 (3)	N3B—C16B—C17B—N4B	1 (3)
C15A—N3A—C16A—C17A	−0.52 (19)	C15B—N3B—C16B—C17B	3 (2)
C15A—N4A—C17A—C16A	0.3 (4)	C15B—N4B—C17B—C16B	−3 (5)
C16A—N3A—C15A—N4A	0.7 (3)	C16B—N3B—C15B—N4B	−5 (3)
C16A—N3A—C15A—C14A	−178.42 (16)	C16B—N3B—C15B—C14B	−178.0 (19)
C17A—N4A—C15A—N3A	−0.6 (4)	C17B—N4B—C15B—N3B	5 (5)
C17A—N4A—C15A—C14A	178.51 (19)	C17B—N4B—C15B—C14B	179 (2)
N6A—C20A—C21A—N5A	−0.1 (3)	N6B—C20B—C21B—N5B	0 (3)
C19A—N5A—C21A—C20A	0.1 (2)	C19B—N5B—C21B—C20B	−5 (2)
C19A—N6A—C20A—C21A	0.2 (3)	C19B—N6B—C20B—C21B	5 (3)
C20A—N6A—C19A—N5A	−0.1 (3)	C20B—N6B—C19B—N5B	−8 (4)
C20A—N6A—C19A—C18A	178.17 (19)	C20B—N6B—C19B—C18B	178 (2)
C21A—N5A—C19A—N6A	0.0 (3)	C21B—N5B—C19B—N6B	8 (3)
C21A—N5A—C19A—C18A	−178.26 (16)	C21B—N5B—C19B—C18B	−178.8 (18)

### Tris(2-methyl-1H-imidazol-3-ium) benzene-1,3,5-tricarboxylate Hydrogen-bond geometry (Å, º)

**Table:**

*D*—H···*A*	*D*—H	H···*A*	*D*···*A*	*D*—H···*A*
N1*A*—H1*A*···O1*A*^i^	0.88 (1)	1.73 (1)	2.6101 (17)	174 (2)
N2*A*—H2*A*···O4*A*^ii^	0.87 (1)	1.83 (1)	2.6888 (18)	169 (2)
C10*A*—H10*C*···O2*A*^i^	0.98	2.42	3.390 (2)	171
C12*A*—H12*A*···O5*A*^ii^	0.95	2.45	3.338 (2)	156
N3*A*—H3*A*···O5*A*^iii^	0.88 (1)	1.76 (1)	2.6309 (16)	172 (2)
N4*A*—H4*A*···O2*A*	0.88 (1)	1.82 (1)	2.682 (2)	170 (2)
C14*A*—H14*B*···O6*A*^iii^	0.98	2.43	3.3867 (19)	165
C17*A*—H17*A*···O3*A*	0.95	2.44	3.3202 (19)	155
N5*A*—H5*AA*···O3*A*^iv^	0.87 (1)	1.75 (1)	2.6131 (17)	173 (2)
N6*A*—H6*A*···O6*A*^v^	0.87 (1)	1.86 (1)	2.713 (2)	167 (2)
C18*A*—H18*A*···O1*A*	0.98	2.63	3.462 (2)	143
C18*A*—H18*C*···O4*A*^iv^	0.98	2.45	3.371 (2)	157
C20*A*—H20*A*···O1*A*^v^	0.95	2.38	3.2993 (19)	164
N1*B*—H1*B*···O1*B*^iv^	0.88	1.64	2.510 (16)	169
N2*B*—H2*B*···O4*B*^v^	0.88	1.75	2.606 (16)	165
C10*B*—H10*D*···O2*B*^iv^	0.98	2.61	3.51 (2)	151
C10*B*—H10*E*···O2*B*^vi^	0.98	2.05	2.85 (3)	137
C12*B*—H12*B*···O5*B*^v^	0.95	2.58	3.475 (15)	157
N3*B*—H3*B*···O5*B*^vii^	0.88	1.66	2.522 (15)	168
N4*B*—H4*B*···O2*B*^iv^	0.88	1.75	2.598 (16)	162
C17*B*—H17*B*···O3*B*^iv^	0.95	2.52	3.442 (16)	164
N5*B*—H5*BA*···O3*B*^i^	0.88	1.65	2.518 (15)	168
N6*B*—H6*B*···O6*B*^ii^	0.88	1.83	2.648 (19)	153

Symmetry codes: (i) −*x*+1, −*y*+1, −*z*+1; (ii) −*x*+1, −*y*, −*z*+2; (iii) *x*−1, *y*+1, *z*; (iv) −*x*+1, −*y*+1, −*z*+2; (v) −*x*+2, −*y*+1, −*z*+1; (vi) *x*+1, *y*, *z*; (vii) −*x*+2, −*y*, −*z*+2.
